# First Gonadal Stages of Bonito, *Sarda chiliensis* (Perciformes: Scombridae) on the Pacific Coast of Northern Chile

**DOI:** 10.12688/f1000research.173271.2

**Published:** 2026-04-24

**Authors:** Renzo Pepe-Victoriano, Marcos Godoy, Karina Kush, Jordan I Huanacuni, Felipe Méndez-Abarca, Luis Espinoza-Ramos, Juan Zenón Resurrección-Huertas, Rodrigo Cortés, Manuel Vergara

**Affiliations:** 1 Facultad de Recursos Naturales Renovables, Área de Biología Marina y Acuicultura, Universidad Arturo Prat, Iquique, Tarapacá Region, 2120, Chile; 2 Núcleo de Investigación Aplicada e Innovación en Ciencias Biológicas, Universidad Arturo Prat, Iquique, Tarapacá Region, 1110939, Chile; 3 Laboratorio de Biotecnología Aplicada, Facultad de Ciencias de la Naturaleza, Universidad San Sebastian, Patagonia, Puerto Montt, 5480000, Chile; 4 Centro de Investigaciones Biológicas Aplicadas, Puerto Montt, 5480000, Chile; 5 Campus Tacna, Universidad Tecnológica del Perú, Tacna, Tacna, 23003, Peru; 6 Grupo de Investigación Acuicultura Sostenible, Universidad Nacional Jorge Basadre Grohmann, Tacna, Tacna, 23004, Peru; 7 Escuela de Ingeniería Pesquera, Universidad Nacional Jorge Basadre Grohmann, Tacna, Tacna, 23004, Peru; 8 Escuela de Ingeniería Acuícola, Facultad de Ingeniería Pesquera, Universidad Nacional Jose Faustino Sanchez Carrion, Huacho, Lima Region, 15135, Peru

**Keywords:** Histological sections, gonads, reproduction, sexual maturity, spawning

## Abstract

**Background:**

The study of the reproductive biology of pelagic fish species, such as ***Sarda chiliensis*** (Bonito), is essential for effective fisheries management and aquaculture development. Overfishing has significantly impacted the population of pelagic fish species, making it crucial to understand their reproductive cycles, including maturity stages and spawning periods, to ensure sustainable exploitation.

**Methods:**

This study provides the first histological characterization of the early gonadal stages of ***Sarda chiliensis*** from northern Chile. A total of 444 specimens were collected from artisanal fisheries between December 2013 and June 2014. Gonadal maturity stages were identified using histological techniques, and a range of microscopic stains (Hematoxylin-Eosin, Van Gieson, and Periodic Acid–Schiff ) were employed to examine the cellular structure of the gonads.

**Results:**

Two key gonadal stages—inactive and previtellogenic—were identified in females, while males exhibited both immature and mature stages. Histological observations revealed distinct characteristics of early gonadal development, including primary oocytes and spermatogonia, with evidence of early vitellogenesis in females. No mature females were observed, and the smallest mature female recorded was 48.5 cm in total length. The gonadosomatic index (GSI) exhibited temporal fluctuations, with a peak observed in February.

**Conclusions:**

This study provides vital histological data on the early gonadal stages of ***Sarda chiliensis*** from northern Chile, offering a baseline for future reproductive studies and aquaculture initiatives. The absence of mature females in the sample highlights the need for broader temporal and spatial sampling to fully characterize the reproductive cycle. These findings are crucial for developing sustainable fisheries management strategies and improving broodstock conditioning for aquaculture.

## 1. Introduction

Overfishing is the primary driver of the decline in pelagic fish stocks. ^1^ To evaluate and manage resources such as *Sarda chiliensis*, it is essential to identify key biological variables, including size at first maturity, spawning periods, and reproductive potential. ^2^ These variables must be determined through accurate assessments of gonadal maturity stages. ^3^ Such studies provide the foundation for developing management and fishery strategies that align with the principles of sustainable exploitation. ^4^

Histology is a powerful tool for examining the microanatomy of gonads, ^5, 6^ playing a crucial role in species management, ^7^ conservation ^8^ and the improvement of domestication success. Initial reproductive studies of *S. chiliensis* were conducted by Barnhart, ^9^ who reported that this species spawns primarily in June off the coast of California. Walford ^10^ observed that the breeding season occurs up to 80 miles offshore, between spring and summer, in northern California. Schweigger ^11^ further documented the breeding period as extending from September to March. Chirinos de Vildoso ^12^ identified peak reproductive activity using a macroscopic gonadal maturity scale, reporting intense gonadal development from October to March. In addition, Chirinos de Vildoso ^12^ performed cytological characterizations of gonadal development, reproductive cycles, and fecundity, applying the macroscopic gonadal maturity scale originally developed by Schaefer and Orange ^13^ for female yellowfin tuna ( *Thunnus albacares*). ^3^

Based on records from all fishing gear types in Malibu, California, in 1978, Collette and Nauen ^14^ reported that the smallest sexually mature *S. chiliensis* (commonly known as bonito) measured between 47 and 53 cm in length. Other studies have indicated that sexual maturity is typically reached at approximately two years of age. ^15^ Research on the spawning period of *S. chiliensis* in Chile ^16, 17^ and Peru ^12^ suggests that spawning begins in September and concludes before April, a pattern corroborated by more recent studies. ^18, 19^

Barrett, ^16^ Serra *et al.*, ^17^ and Chirinos de Vildoso ^12^ based their conclusions on gonadosomatic indices (GSIs) and microscopic examinations of unstained gonads, which were later supported by histological analyses conducted by Goldberg and Mussiett. ^20^ These authors observed that in northern Chile, *S. chiliensis* exhibits a seasonal reproductive cycle typical of temperate fish species, characterized by a spawning phase lasting approximately half the year, followed by a non-reproductive period. This cycle coincides with periods of high food availability for larvae. Goldberg and Mussiett ^20^ partially confirmed Barrett’s ^16^ findings, reporting that females measuring approximately 51 cm in total length (TL) were mature, with the smallest mature female measuring 48.5 cm—slightly smaller than Barrett’s ^16^ original estimate.

Temporal differences in peak spawning periods observed in the study area have also been documented in other pelagic species of the Chile-Peru region. For instance, the anchoveta ( *Engraulis ringens*) exhibits peak reproductive activity from July to February in Chile ^21^ and from September to March in north-central Peru. ^22, 23^ Similarly, *Trachurus murphyi* reproduces between September and December, with peak GSI values recorded in November. ^24^

Although Goldberg and Mussiett ^20^ defined four stages of gonadal maturity based on the size and characteristics of the ovaries and testes, they did not employ histological techniques due to the absence of simplified criteria for identifying early stages. ^25^ Such information is essential in fisheries biology, as recognizing the microscopic features of early oocyte development ^26^ is critical for accurately determining reproductive status. Because this species lacks external sexual differentiation, histological analysis is necessary. Previous studies on *S. chiliensis* in Peru and Chile have based mainly on macroscopic descriptions of gonadal development or indices such as the gonadosomatic index, without providing detailed histological evidence of early reproductive stages. Despite the ecological and commercial importance of *Sarda chiliensis* in the southeastern Pacific, detailed histological descriptions of early gonadal development stages remain scarce for populations inhabiting the northern Chilean coast. Histological characterization of reproductive tissues provides valuable baseline information for understanding reproductive processes in fish populations, particularly in regions where long-term reproductive monitoring is limited. Therefore, the aim of this study was to describe the early gonadal developmental stages of *Sarda chiliensis* through histological examination of specimens collected from fishery landings in northern Chile. Due to the temporal and spatial scope of the sampling design, this study focuses on the characterization of early gonadal development rather than on a complete assessment of the reproductive cycle of the species.

## 2. Methods

This study did not involve experimental procedures on tissues, cell lines, or human subjects. All methodologies complied with current Chilean legislation, specifically Law 20.380 on Animal Protection.

The fish were not collected exclusively for this research; therefore, no additional ethical approval was required. Collection procedures adhered to the regulations established by the Servicio Nacional de Pesca y Acuicultura (SERNAPESCA) of Chile. Specimens were purchased at the local fishing terminal after being recently caught by artisanal fishermen, in accordance with the guidelines of SERNAPESCA and the Ministry of Health.

It should also be noted that *S. chiliensis* is neither subject to catch restrictions nor listed as a protected or endangered species.

**Figure 1.**
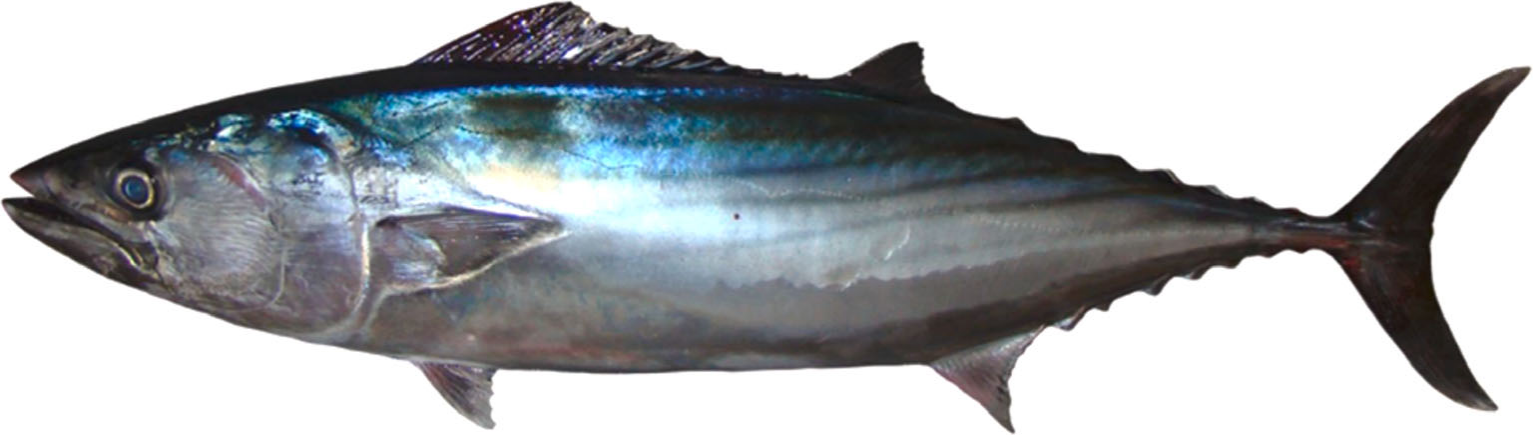
Adult specimen of *Sarda chiliensis.* The species has a fusiform body with a metallic blue sheen on the flanks and five to nine dark, oblique stripes extending from the dorsal area toward the ventral side (adapted from Pepe-Victoriano *et al*. ^19^).

*S. chiliensis* ( Figure 1) is an epipelagic and neritic species, ^14^ characterized by a fusiform body with metallic dark-blue coloration and five to nine dark, oblique stripes extending from the dorsal to the ventral side. ^27^ It possesses a large mouth, conical teeth, and prominent round eyes. ^28^ This species is distributed from Máncora, Peru—just south of the Gulf of Guayaquil—southward to Talcahuano in Chile.

### 2.1 Capture and study area

A total of 444 *S. chiliensis* specimens were collected using a seine net (locally referred to as *boliche*) along the coastal margin of the Chanavayita sector (20°42′16″S, 70°11′16″W). Chanavayita is located approximately 60 km south of Iquique, in the Tarapacá Region. Samples were obtained by artisanal fishermen between December 2013 and June 2014. This site was chosen because it constitutes a primary fishing ground for this species in the region’s major ports (Arica, Iquique, and Antofagasta), particularly along the stretch between Iquique and Mejillones. ^16^

### 2.2 Measurements and laboratory observations

Once obtained from artisanal fishermen, the specimens were transported to the laboratory for morphometric analysis. TL and standard length (SL) were measured to the nearest 0.01 cm using an ichthyometer. Total weight (TW) and gutted weight (GW) were recorded to the nearest 0.1 g using a digital scale (Ohaus, Ranger 3000 Series, Model R31P3, 3000 g × 0.1 g). Sex identification was conducted by making a longitudinal incision from the mandibular symphysis to the anal orifice to expose the gonads.

### 2.3 Gonadal processing

The fish were transported to the laboratory 7 hours post-harvest and processed immediately to minimize tissue degradation. The gonads were fixed using 8% buffered formalin or Bouin-Hollande fixative in a ratio of at least 10:1 (fixative volume:tissue volume). Samples remained in fixator for 24–36 h depending on the size of the gonads, after which they were transferred to 70% ethanol for storage prior to dehydration and inclusion. These conditions were applied to reduce the risk of histological alterations and ensure optimal preservation of cell structures. In the laboratory, three subsamples were taken from each gonad, embedded in Paraplast paraffin, and sectioned at a thickness of 7–12 μm using a rotary microtome (Microm Leitz Wetzlar 13-13). The sections were subsequently deparaffinized and dehydrated through a graded ethanol series. Histological staining was carried out using Hematoxylin-Eosin (H&E), Van Gieson, and Periodic Acid–Schiff (PAS) stains for microscopic examination. Histological observations were performed with an Olympus CX31 optical microscope equipped with a Nikon digital camera. A total of 10 slides per sample were analyzed.

### 2.4 Maturity status

Gonadal development stages were described following the standardized terminology proposed by Brown-Peterson *et al*., ^29^ which is widely used in modern studies of fish reproductive biology was applied. This scale distinguishes six stages: inactive, previtellogenic, vitellogenic, mature, hydrated, and spawning (the latter characterized by the presence of post-ovulatory follicles). Male specimens were classified as either immature or mature.

### 2.5 Statistical analyses

Statistical analyses were performed in RStudio version 2024.09.0+375 (RStudio, Inc., Washington, DC, USA). Maturity stages were tested for normality using the Anderson–Darling test and for homogeneity of variances using Bartlett’s test. ^30^ The non-parametric Mann-Kendall correlation test (Kendall’s Tau) was applied to assess the relationship between the GSI and temperature. Pearson’s correlation test was used to evaluate the relationship between Fulton’s condition factor ( *k*), weight, and length across different maturity stages. All graphs were generated with the *ggplot2* package in RStudio and are presented as mean ± standard deviation (SD). Given the limited temporal coverage of the dataset, these analyses should be interpreted cautiously and are intended primarily to identify potential patterns that may guide future research rather than to establish definitive biological relationships.

## 3. Results

The number of specimens captured, along with their weight and length ranges, is presented in Table 1. The numbers of mature female and male specimens are provided in Table 2.

**Table 1.** Morphometric parameters and frequency distribution of *Sarda chiliensis* specimens captured off the coast of Chanavayita, Chile. TL = Total length; TW = Total weight.

Sex	Number	%	TL (cm, mean ± SD)	TL Range (cm)	TW (g, mean ± SD)	TW Range (g)
Females	224	50.45	43.5 ± 1.5	40.3 – 48.8	1,116.1 ± 105.6	826 – 1,437
Males	205	46.17	45.5 ± 1.8	41.2 – 50.4	1,222.8 ± 131.3	881 – 1,617
Undetermined	15	3.38	40.0 ± 0.9	39.1 – 41.5	761 ± 31	701 – 805
Total	444	100				

**Table 2.** Maturity stages of *Sarda chiliensis* specimens, classified by sex and developmental stage. TL = Total length; TW = Total weight.

Sex	Maturity stage	Number of specimens	Percentage per sex (%) *	TL (cm, mean ± SD)	TW (g, mean ± SD)
Females	Previtellogenic	31	13.8	46.1 ± 1.2	1,218 ± 93.1
	Immature	193	86.2	43.1 ± 1.1	1,099.7 ± 98.3
Males	Mature	104	50.7	46.8 ± 1.1	1,247.2 ± 126.1
	Immature	101	49.3	44.1 ± 1.3	1,197.6 ± 132.5
Total		429			

### 3.1 Gonadosomatic Index (GSI)

**Figure 2.**
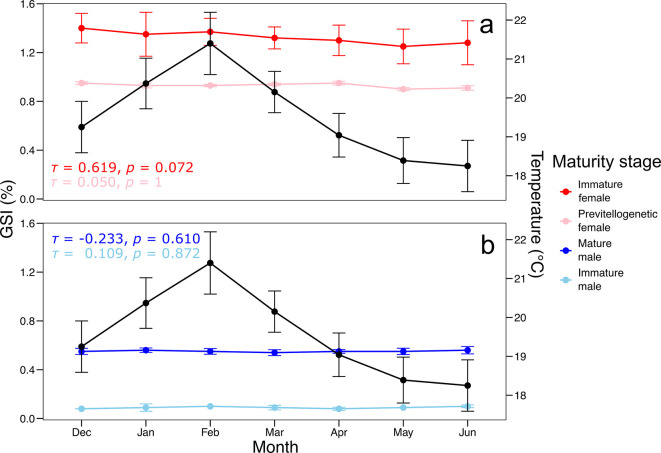
Monthly variation in the GSI (%) of *Sarda chiliensis* by gonadal maturity stage and its relationship with water temperature on the northern coast of Chile. The black line represents mean monthly water temperature (°C), while the colored lines indicate GSI values for each maturity stage in (a) females and (b) males.

The results demonstrated that the GSI exhibited marked monthly variations between December and June across the different gonadal maturity stages of *S. chiliensis* ( Figure 2). Inactive females displayed a peak GSI in February (1.4 ± 0.1%), whereas previtellogenic females maintained consistently low and stable values (0.7 ± 0.05%). Mature males exhibited a slight increase in GSI in February (0.6 ± 0.05%), while immature individuals consistently showed low values (< 0.4%).

Kendall *’*s tau correlation analysis indicated a non-significant positive correlation between GSI and temperature for inactive females (τ = 0.619, p = 0.072) *,* with no significant relationships observed for the other maturity stages (p > 0.6).

### 3.2 Fulton’s condition factor (k)

**Figure 3.**
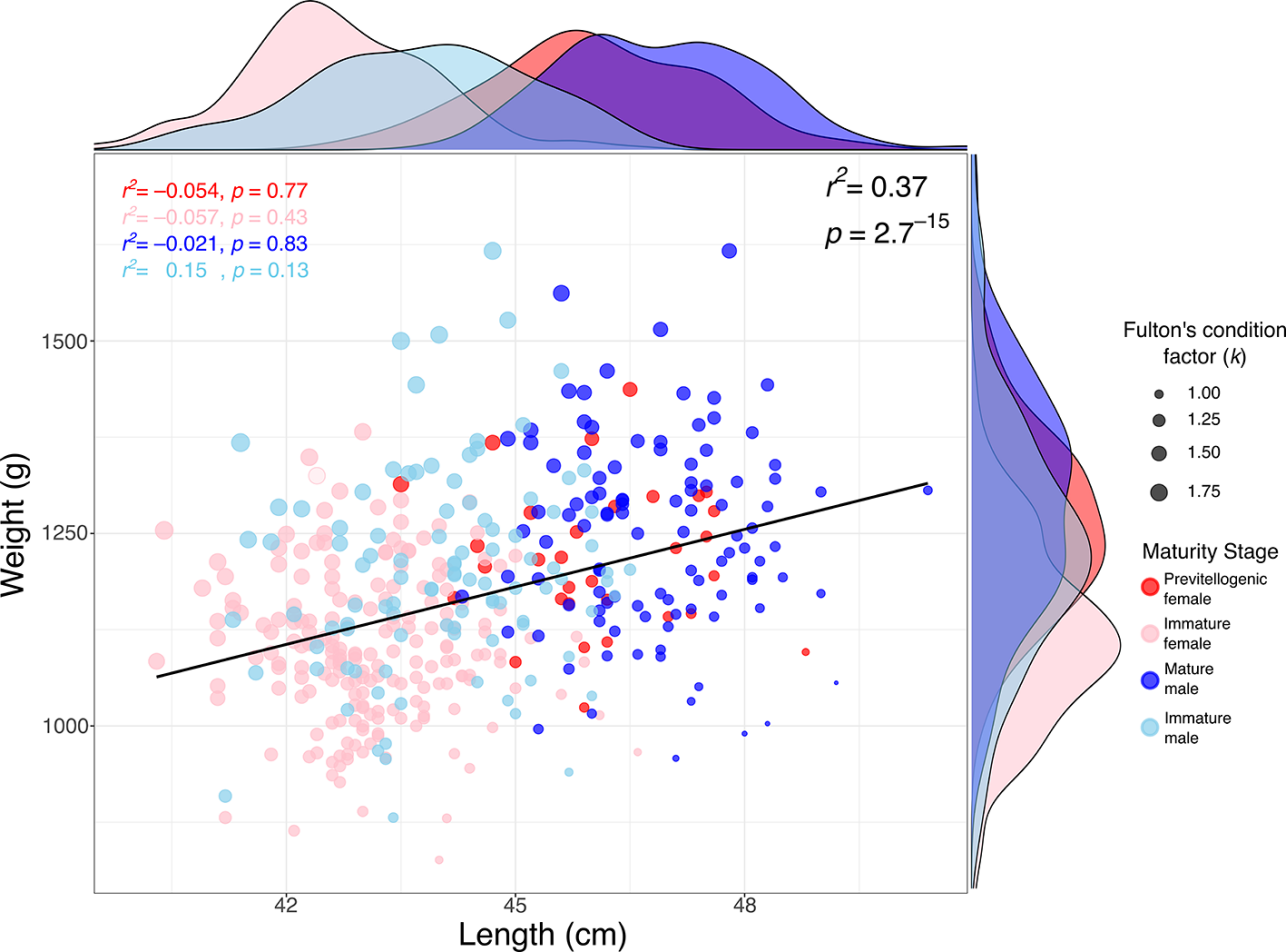
Relationship between weight and length in bonito ( *Sarda chiliensis*) across maturity stages, and Fulton’s condition factor ( *k*), along the Pacific coast of northern Chile.

The relationship between weight and length in *S. chiliensis* is presented in Figure 3. A significant positive correlation was observed between weight and TL (r ^2^ = 0.37; p = 2.7 × 10 ^-15^), indicating that greater lengths are generally associated with higher weights. Analysis by maturity stage showed that mature individuals (primarily males) tended to exhibit higher weights and lengths (see marginal densities), *whereas* immature individuals and previtellogenic females were clustered within lower weight and length ranges. Furthermore, Fulton *’*s condition factor (k), represented by the size of the points in the figure, was generally higher in mature individuals, suggesting a better overall body condition in this group.

### 3.3 Gonadal histological description in females

Based on the analyzed samples, only two maturation stages were identified: inactive and previtellogenic. To supplement the histological description of the gonadal stages, we summarized the primary germ cell types observed in both male and female specimens. For each cell type, the associated developmental stage, approximate diameter, distinctive morphological features, and corresponding figure references are provided ( Table 2).

The main germ cell types observed in both sexes, together with their developmental stage, diameter, morphological characteristics, and figure references, are summarized in Table 3.

**Table 3.** Summary of germ cell types identified in *Sarda chiliensis*, with corresponding developmental stages, cell diameters, morphological characteristics, and figure references.

Cell type	Stage	Diameter (μm)	Morphology	Label in figures
O1 – Primary oocyte	Inactive (Female)	60–100	Central nucleus, basophilic cytoplasm no follicular layer	Figure 4a, 4b
PvO – Previtellogenic oocyte	Previtellogenic (Female)	250–300	Large oocyte with centrally located nucleus and nucleoli located at the periphery of the nucleus, cytoplasm slightly basophilic	Figure 6a, 6b
SG – Spermatogonia	Immature (Male)	-	Large round cells located at the periphery of seminiferous lobules	Figures 7– 8
SZ – Spermatozoa	Mature (Male)	-	Cells with condensed nuclei located in seminiferous ducts	Figure 8a–f

**Figure 4.**
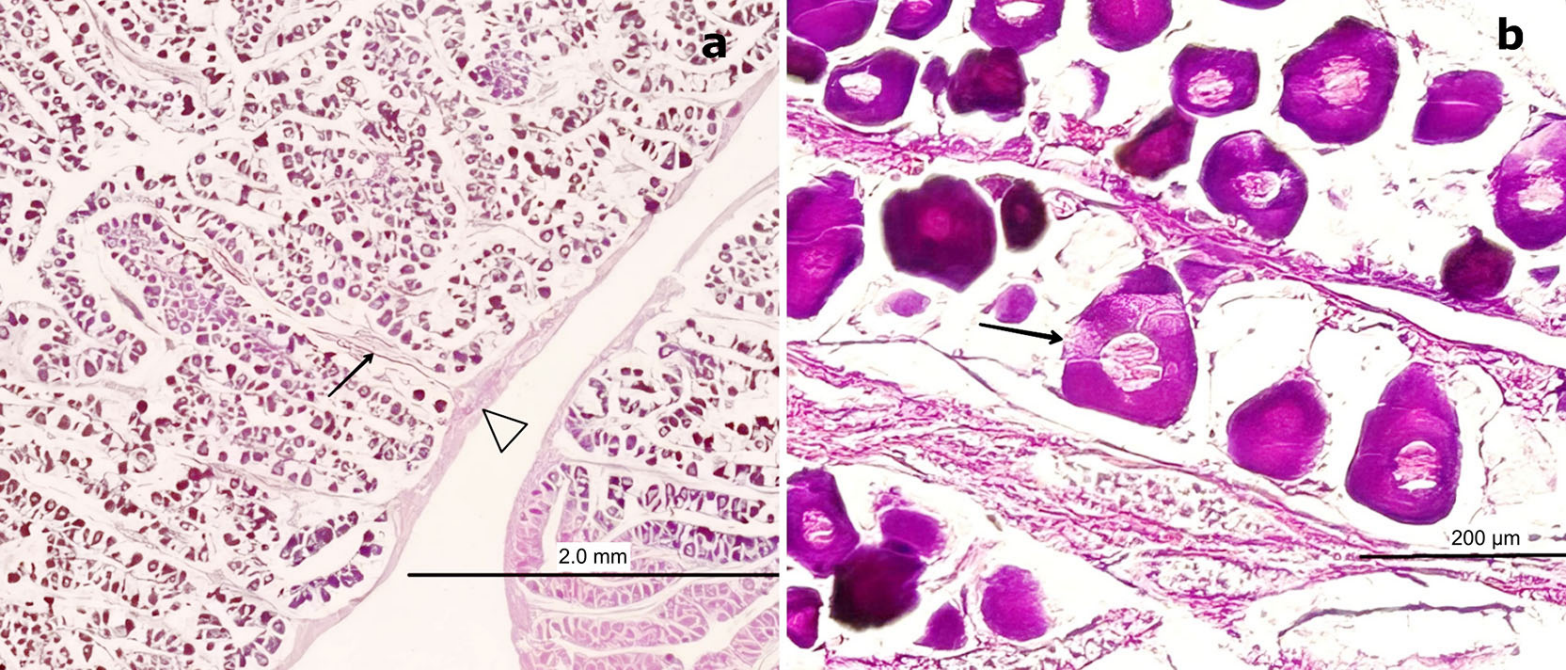
Ovarian tissue of *Sarda chiliensis* at the inactive maturation stage. (a) Peripheral region of the ovary with a thin layer of connective tissue (CT, white arrowhead) extending toward the medullary zone (black arrow). Scattered primary oocytes (O1) with centrally positioned nuclei (NO) are visible within the stromal tissue. (b) Higher magnification of O1 oocytes, showing their spherical outline, vesicular nuclei with one to five nucleoli (NO) adjacent to the nuclear membrane, and absence of follicular cells (FC). In some oocytes, initial signs of follicular arrangement can be observed. Hematoxylin-eosin stain. Scale bar = 200 μm. Magnifications: (a) 40×, (b) 400×.

H&E staining ( Figure 4) revealed that the outer portion of the gonad is composed of a thin layer of moderately dense CT, consisting of collagen fibers oriented in a single direction. These fibers extend into the medullary zone of the gland as thinner bundles, forming lobules. Within these lobules, oocytes at the inactive maturation stage (also referred to as the primary maturation stage) were observed.

At this stage, oocytes are small (60–100 μm), round to ovoid in shape, and lack follicular cells in their membrane. These primary oocytes (O1) are centrally nucleated and surrounded by CT, with clearly visible nucleoli (NO) under high magnification. Internally, they exhibit a well-defined vesicular nucleus located centrally. At 400× magnification, 1–5 NO can be observed along the nuclear membrane.

**Figure 5.**
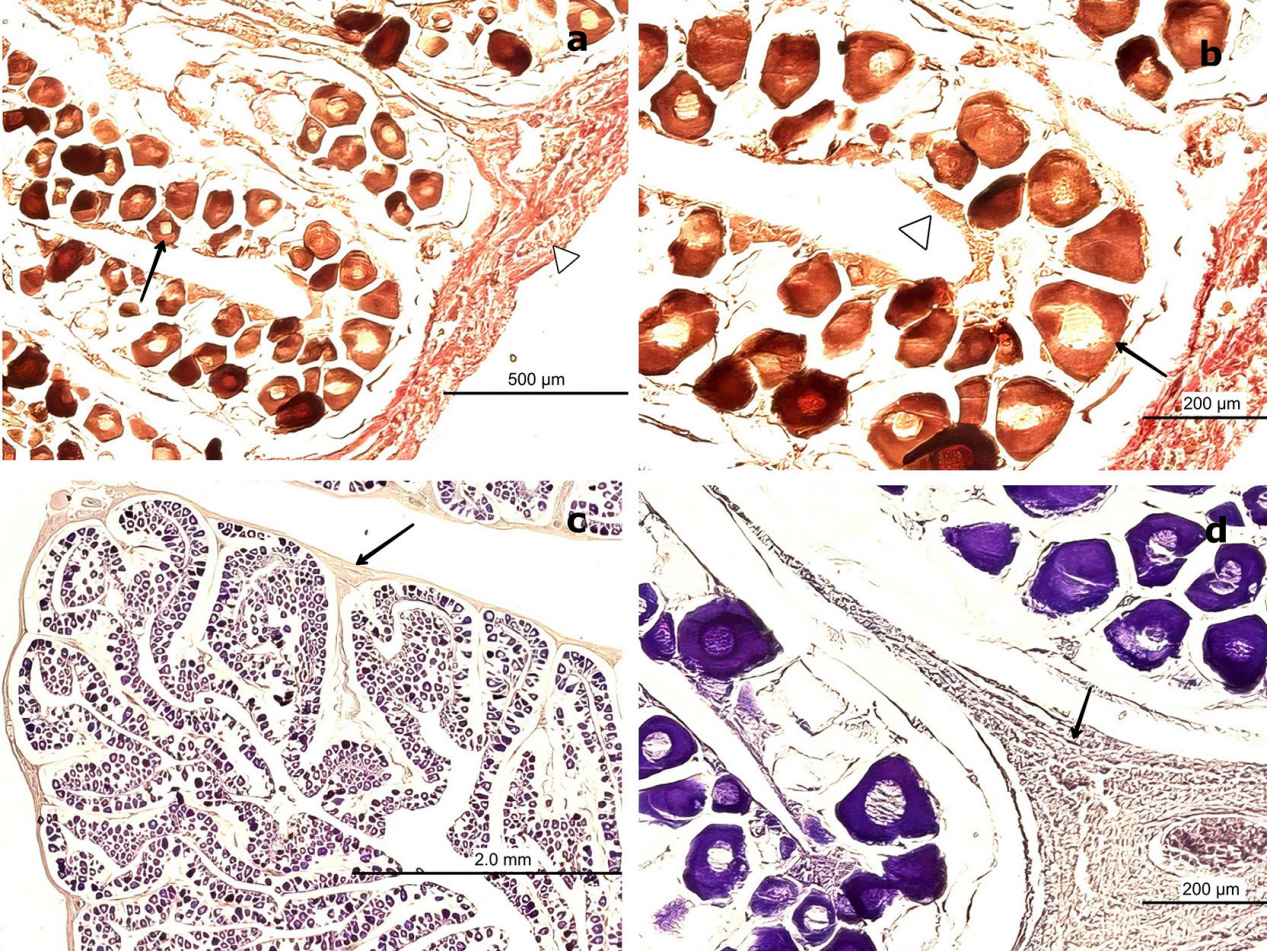
Ovarian tissue of *Sarda chiliensis* at the inactive maturation stage. (a, b) Van Gieson stain showing primary oocytes (O1) with a rounded outline and centrally located nuclei (NO, black arrow), surrounded by connective tissue (CT, white arrowhead) and sparse follicular cells (FC). (c, d) Periodic acid–Schiff (PAS) stain displaying oocytes within the cortical (ZC) and medullary (ZM) zones, highlighting nuclear details (NO) and the interstitial connective matrix (CM, black arrow). Magnifications: (a) 200×, (b) 400×, (c) 200×, (d) 400×.

Van Gieson staining ( Figure 5a, 5b) revealed the presence of blood vessels (BV), including arteries and veins, in the peripheral region of the gland. In contrast, PAS staining ( Figure 5c) produced a negative reaction in the CT surrounding the gland, indicating the absence of mucopolysaccharides or glycoproteins ( Figure 5d). Conversely, a positive PAS reaction was detected in the oocyte nucleoplasm, confirming the presence of 1–5 NO along the nuclear membrane, consistent with the results obtained using H&E staining.

3.3.1 Previtellogenic maturation stage

At this stage, the gonad exhibits no significant structural changes in the stromal tissue regarding its distribution and proportion relative to the parenchyma. H&E staining reveals no cytological alterations in the peripheral region of the medullary zone.

**Figure 6.**
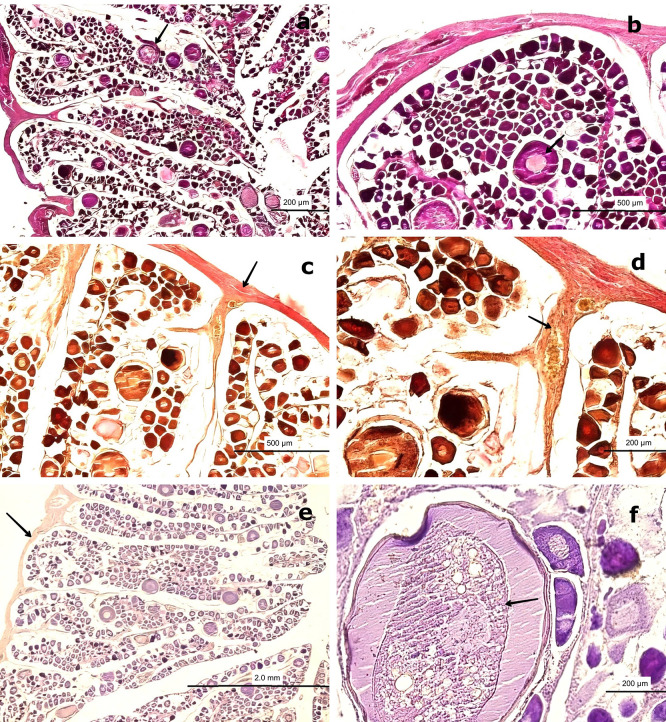
Histological features of the female gonads of *Sarda chiliensis* at the previtellogenic maturation stage. (a) Previtellogenic oocytes (PvO, black arrow) with a round to ovoid outline and increased size (250–300 μm), surrounded by connective tissue (CT). Hematoxylin-eosin (H&E) stain. (b) Higher magnification of PvO showing peripheral vesicles (V) and a centrally located nucleus (NO), characteristic of early vitellogenesis. H&E stain. (c,d) Van Gieson stain revealing increased collagen fibers (CF) in the medullary and peripheral zones of the gonad (black arrow), as well as the lobular structure and CT surrounding the oocytes. (e) Negative reaction of the connective tissue in the Periodic acid–Schiff (PAS) stain (black arrow), confirming the absence of mucopolysaccharides. (f ) Positive PAS reaction in early primary oocyte (PO) adjacent to the PvO, indicating the presence of glycoproteins (black arrow). Magnifications: (a) 40×, (b) 400×, (c) 200×, (d) 400×, (e) 100×, (f ) 400×.

The oocytes increase in size (250–300 μm) and are round to ovoid in shape, with centrally located ovoid nuclei. The CT surrounds these previtellogenic oocytes (PvO), which display early cytoplasmic vesicles (V) characteristic of primary vitellogenesis. Four to five NO are observed at the nuclear periphery, and their positive PAS reaction indicates the presence of glycoproteins. The NO, measuring approximately 2–5 μm, increase in number to four or five and remain attached to the nuclear membrane. The cytoplasm becomes more homogeneous and less basophilic compared to the previous stage. Surrounding the nucleus, vacuoles or spherical vesicles indicative of primary vitellus—characteristic of the previtellogenic stage—are present ( Figure 6a, 6b).

Van Gieson staining reveals an increase in collagen fibers, which are oriented uniformly within the CT surrounding the gland. This change is consistent with the histophysiological development of the gland, as both the oocytes and the gonad itself increase in size. An increased presence of BV, including veins and arteries, is also evident.

Analysis of the peripheral region of the gonad using Van Gieson staining revealed clearly visible BV, including arteries and veins. PAS staining of the CT surrounding the oocytes showed a negative reaction, confirming the absence of mucopolysaccharides or glycoproteins. However, at 400× magnification, a diffuse positive PAS reaction was observed in the nucleolus, warranting further investigation to precisely determine its localization ( Figure 6e, 6f).

### 3.4 Male histological gonadal description

**Figure 7.**
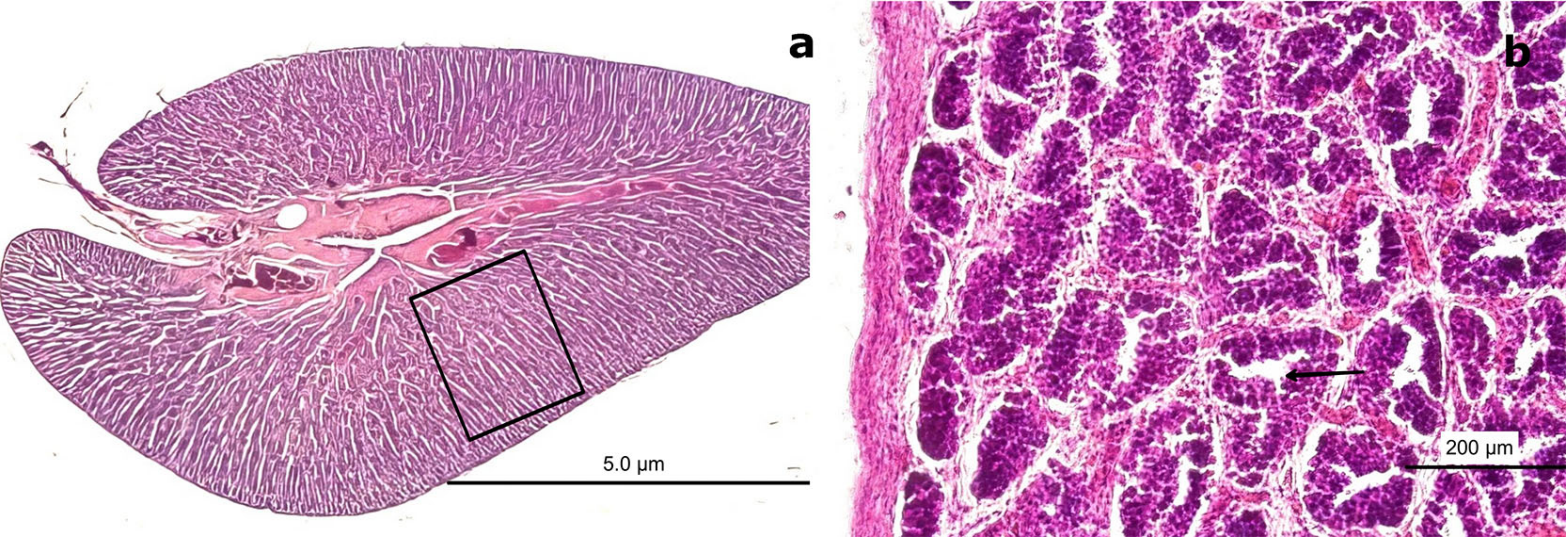
Histological characteristics of male gonads of *Sarda chiliensis* at the immature stage. (a) Low-magnification image showing seminiferous cords (SC) arranged concentrically and composed exclusively of spermatogonia (SG, boxed area). No spermatozoa (SZ) are present. The surrounding connective tissue (CT) is also visible. (b) Higher-magnification image of SC revealing densely packed SG with large, round nuclei and the absence of mature germ cells (SZ) or a defined lumen (L, black arrow). Hematoxylin-eosin (H&E) stain. Magnifications: (a) 40×, (b) 200×.

In males, spermatogonia (SG) and spermatozoa (SZ) were identified and characterized as detailed in Table 3. Among the mature males, 50 individuals exhibited a high abundance of SZ within the saccules or follicles, as well as in the mesonephric ducts (MSD). However, 14 specimens displayed signs of immaturity in the cortical zone of the gland ( Figure 7). Forty males showed incomplete maturation, as evidenced by concentrically arranged seminiferous cords (SC) composed entirely of SG. These cells were embedded within a CT matrix and possessed large, round nuclei. The developing lumen and cords lacked SZ, indicating an immature state ( Figure 7a, 7b).

3.4.1 Immature stage

**Figure 8.**
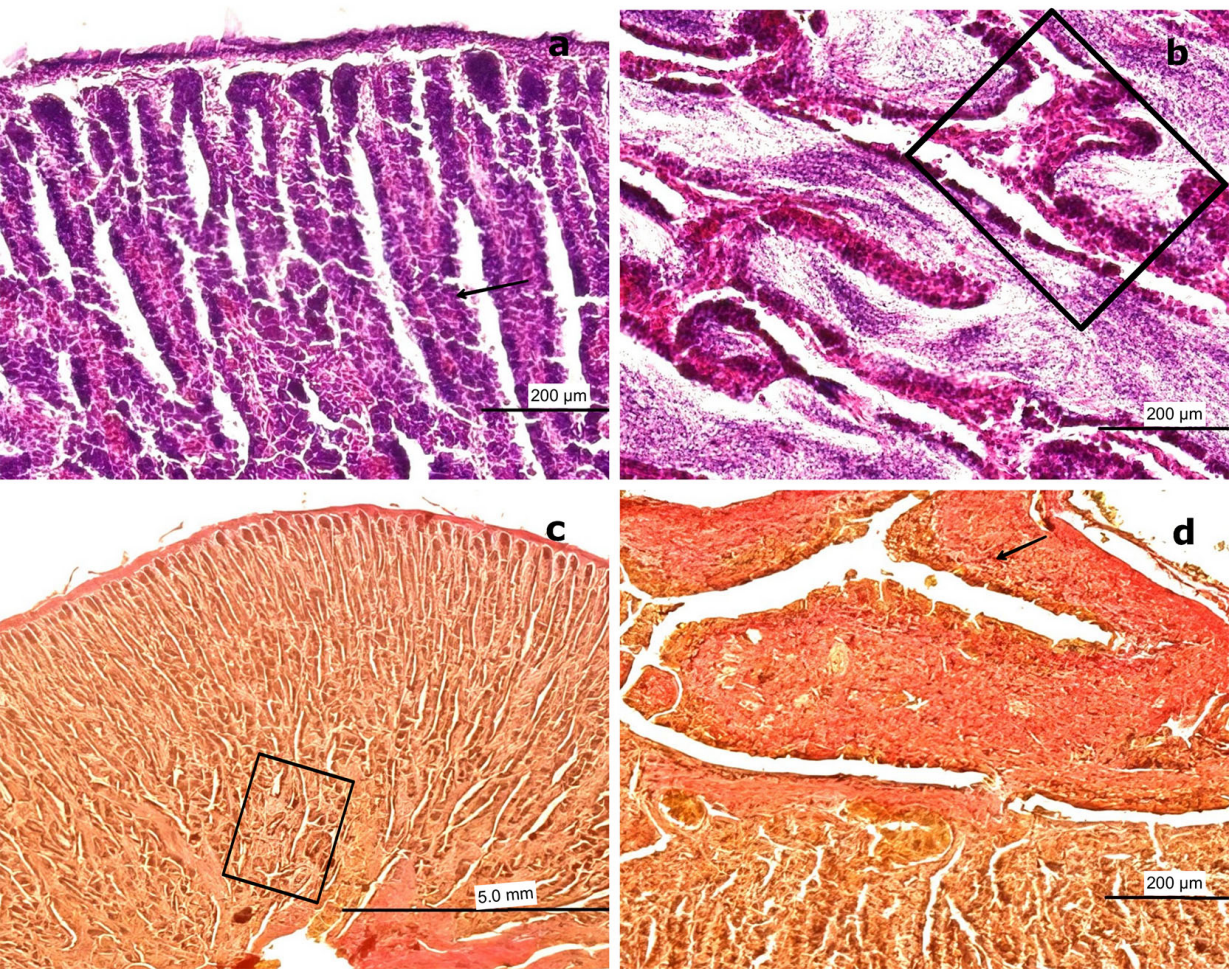
Histological features of male gonads of *Sarda chiliensis* at the immature stage. (a) Immature seminiferous duct (SD) lacking sperzoa (SZ) and spermatocytes (ST) in the lumen (black arrow), indicating a non-functional spermatogenic stage. (b) SD with scattered SZ in the lumen (boxed area), representing early signs of spermatogenesis. H&E stain. (c) Van Gieson stain of a central SD (boxed area) surrounded by connective tissue (CT), showing multiple small arterioles (AV) and venules (VN). (d) Higher magnification of the vascular region highlighting AV and VN (black arrow) matoencircling the SD. Van Gieson stain. Magnifications: (a) 40×, (b) 200×, (c) 40×, (d) 200×.

Immature gonads were noticeably smaller than mature ones. The outer layer consisted of CT that extended into the medullary zone. Both the cortex and medulla contained continuous cellular cords that lacked SZ ( Figure 8a). Histological examination revealed no evidence of luminal development, and the seminiferous ducts (SD) were devoid of SZ and spermatocytes (ST) ( Figure 8a). The onset of spermatogenesis was indicated by scattered SZ in the lumen of certain ducts, reflecting initial spermatogenic activity ( Figure 8b). The structural organization necessary for testicular function was highlighted by vascular elements, including arterioles (AV) and venules (VN), which were observed surrounding the SD and embedded within the CT ( Figure 8c, 8d).

The central SD contained small BV, including AV and VN, embedded within the surrounding CT ( Figure 8c, 8d). This vascular arrangement plays a crucial physiological role by supplying nutrients through the vasa vasorum.

3.4.2 Mature stage

**Figure 9.**
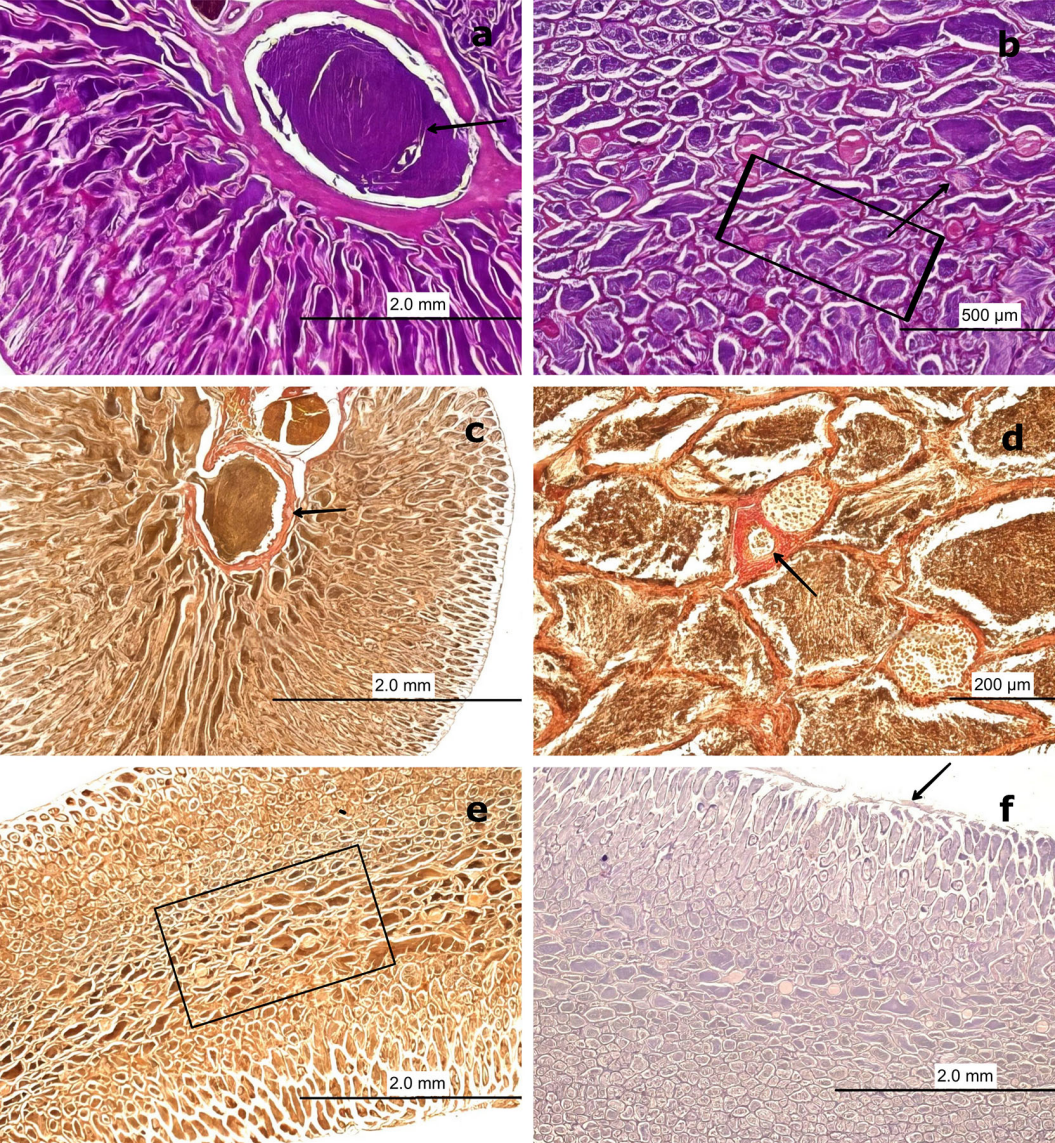
Histological features of male gonads of *Sarda chiliensis* at the mature stage. (a) General view of the gonad showing several spermatocyst (SS) containing developing spermatozoa (SZ, black arrowhead). (b) Advanced spermatogenesis indicated by dense clusters of SZ within SS at higher magnification (white arrowhead). (c) Van Gieson stain highlighting blood vessels (BV) surrounded by connective tissue (CT) and mesonephric ducts (MSD) with associated SS. (d) Higher magnification of SS within MSD (black arrow), with adjacent CT and BV clearly visible. (e) Longitudinal section of CT showing MSD with vascular structures (BV) and embedded SS (boxed area). (f ) PAS stain showing a negative reaction in the CT (boxed area), confirming the absence of glycoproteins and mucopolysaccharides (M). Magnifications: (a) 40×, (b) 400×, (c) 40×, (d) 400×, (e) 40×, (f ) 100×.

Among the mature gonads, 20 exhibited slight immaturity at the cortical level, surrounded by an albuginea composed of CT and collagen fibers. Histological analysis of the mature gonads revealed a well-developed outer capsule (albuginea) made of collagen fibers and CT, which extended into the medullary zone and formed concentrically arranged spermatocyst (SS) ( Figure 9a). Advanced spermatogenesis was indicated by the abundance of SZ found within the SS ( Figure 9b). MSD filled with SZ and encircled by BV were observed in the central region of the gonad. These ducts were lined with smooth muscle fibers and simple squamous epithelium ( Figure 9c–e). The structure of these ducts resembles the epididymal architecture seen in higher vertebrates. The absence of mucopolysaccharides (M) or glycoproteins was confirmed by a negative PAS reaction in the surrounding CT ( Figure 9f). This configuration indicates a well-organized and functionally effective spermatogenic architecture in fully mature males.

Figures 9a and 9b provide an overall view of a mature gonad, clearly showing the SS. Figure 9c highlights BV–AV and VN, which are essential for cellular nutrition. Figures 9d and 9e present a longitudinal section of the gonad, illustrating the structure of the saccules and the presence of BV.

In the central region of the gonad, MSD composed of smooth muscle interspersed with collagen fibers and lined by simple squamous epithelium were observed. These ducts contained a large quantity of SZ, resembling the epididymal function observed in higher vertebrates ( Figure 9e). At 400× magnification, globose cells characteristic of early spermatogenesis were visible in one half of the saccule, while the other half contained mature SZ ( Figure 9d).

### 3.5 Exploratory statistical analysis

The statistical correlations between gonadosomatic index, condition factor, and sea surface temperature were not significant. These analyses were conducted with an exploratory purpose, aiming to identify potential patterns and generate preliminary hypotheses about the reproductive dynamics of *S. chiliensis.* Given the descriptive and temporally limited nature of this study, these statistical outcomes should not be interpreted as confirmatory evidence but rather as a first step to guide more comprehensive analyses in future research.

## 4. Discussion

It is important to note that the present study focused exclusively on the early gonadal stages observed in *Sarda chiliensis*. Consequently, the findings should be interpreted as a descriptive characterization of initial gonadal development rather than as a complete representation of the species' reproductive cycle. Additional studies covering a broader temporal range and including mature females would be necessary to fully describe the spawning dynamics and reproductive cycle of this species in the study region.

Chirinos de Vildoso ^12^ states that the maturation process is slow and influenced by secondary factors such as feeding ^31^ and morphocytological development. Additionally, gonadal maturation does not occur simultaneously in all specimens.

Unlike previous research that relied predominantly on gross gonadal scales or indirect indices, our study provides the first histological description of the early gonadal stages of *S. chiliensis* from northern Chile. This approach allows for more precise identification of germ cell types and reproductive phases, which is essential for refining species maturity scales. By documenting these stages under the microscope, our work establishes a reliable benchmark for fisheries biology, with direct applications for both the management of wild populations and the development of broodstock conditioning protocols in aquaculture.

Sampling was conducted between December and June of a single year, which certainly limits the possibility of reconstructing the complete reproductive cycle of *S. chiliensis.* We acknowledge this temporal restriction and highlight it as an important limitation of the study. Nevertheless, this period coincides with one of the phases in which artisanal fishing activity is most concentrated in northern Chile, which allowed us to obtain representative specimens from the main landing site of the resource. Therefore, this work should be understood as a preliminary, localized approach that provides baseline information for future research with longer duration and broader spatial coverage.

Regarding the sex ratio of the captured specimens, there was an approximately equal proportion of males and females. ^32^ This outcome may be attributed to the absence of mature females, which reduces the need for courtship behaviors—behaviors that might otherwise differentiate mature males from immature ones. Although Magnuson and Prescott ^33^ assert that pelagic fish generally do not exhibit courtship, they observed such behavior in *S. chiliensis lineolata,* suggesting the presence of temporary courtship and mating habits. Pairs of *S. chiliensis lineolata* were observed engaging in sequential behaviors aimed at the simultaneous and adjacent release of gametes, with males swimming in tandem while females followed a circular trajectory. The absence of mature females in the analyzed samples may be related to spatial or seasonal segregation patterns. In many pelagic fish species, reproductive individuals may migrate to specific spawning areas or occupy habitats different from those exploited by local fisheries. Therefore, it is possible that mature females of *S. chiliensis* occur in other areas or during periods not covered by the present sampling. Future studies incorporating wider spatial and temporal sampling would help clarify the reproductive distribution patterns of this species.

The predominance of females in inactive or previtellogenic stages during this period may be associated with suboptimal thermal conditions necessary to initiate or complete ovarian maturation ( Table 4). This pattern indicates a potential **thermal sensitivity in the reproductive physiology** of the species, where minor variations in SST could delay vitellogenesis or alter the timing and spatial distribution of spawning activity. In this context, SST in the Chanavayita region may act as an **environmental modulator** of the local reproductive cycle of *S. chiliensis*, highlighting the importance of integrating oceanographic monitoring with reproductive studies in future research.

**Table 4.** Gonadal development and spawning characteristics of *Sarda chiliensis* and related species.

Species	Type of gonadal development	Gonadal maturity phases	Optimal temperature for spawning	Gonad size at spawning	Spawning frequency	Source
*Sarda chiliensis*	Sequential gonadal development with well-defined maturity phases	Pre-maturation, maturation, spawning, and post-spawning phases	18–20°C	Relatively large gonads, with high fecundity	Seasonal spawning, once a year	^18, 34^
*Thunnus albacares*	Cyclic gonadal development with a single spawning period per season	Pre-maturation, maturation, and post-spawning phases with seasonal spawning	26–31°C	Moderately sized gonads, lower fecundity	Seasonal spawning in warm water areas, once a year	^35, 36^
*Sarda orientalis*	Sequential gonadal development with a well-defined seasonal spawning	Pre-maturation, maturation, spawning, and post-spawning phases	24.7–28°C	Large gonads, with high fecundity	Seasonal spawning, once a year	^37, 38^
*Katsuwonus pelamis*	Cyclic gonadal development with multiple maturity phases throughout the year	Maturation, spawning, and post-spawning phases, with a higher spawning frequency	23–31°C	Medium-sized gonads, with high fecundity	Continuous spawning throughout the year	^39, 40^

### 4.1 Gonadosomatic Index (GSI)

The GSI values showed fluctuations among the sampled months; however, due to the limited temporal coverage and sample distribution, these observations are presented as descriptive patterns and should not be interpreted as evidence of statistically supported temporal variation suggests a reproductive peak in February, consistent with previous studies on related species of the genus *Sarda.* ^41, 42^ This peak appears to be primarily driven by inactive yet maturing females, which may indicate a synchronous reproductive strategy within the population. The absence of a significant correlation between GSI and temperature suggests that gonadal maturation may be influenced by other environmental or endogenous factors, such as photoperiod or body condition. ^20, 43^ Reproductive cycles in pelagic fishes are often influenced by environmental variability, including temperature fluctuations, food availability, and oceanographic processes. Although the present study recorded environmental parameters during the sampling period, the temporal coverage of the dataset was insufficient to evaluate potential synchrony between environmental variability and spawning periodicity in *S. chiliensis*. Future studies incorporating multi-year sampling and continuous environmental monitoring would provide a more comprehensive understanding of the environmental drivers of reproduction in this species.

These findings provide evidence of reproductive seasonality in *S. chiliensis* along the northern coast of Chile and underscore the importance of such data for the effective management and conservation of the species. The relationship between feeding activity and reproductive development can provide insights into the energetic allocation strategies of fish species. In the present study, gastrointestinal indices were not systematically recorded during sampling, which prevented evaluation of potential relationships between feeding intensity and gonadal development. Incorporating trophic indicators in future studies would allow further exploration of the interaction between feeding dynamics and reproduction.

### 4.2 Fulton’s condition factor (k)

The positive relationship observed between weight and length (r ^2^ = 0.37) in *S. chiliensis* is consistent with typical allometric growth patterns in teleosts and aligns with previous findings in congeneric species, such as *Sarda*, where similar relationships have been reported. ^44– 46^ Variations in weight and length across maturity stages indicate that mature individuals—particularly males—tend to exhibit greater somatic development and enhanced body condition, as reflected by Fulton’s condition factor. This pattern suggests increased energetic investment in gonadal maturation, a trend commonly observed in pelagic fish species. ^42^

These results highlight the importance of considering both maturity stage and body condition in population assessments and broodstock management of *S. chiliensis* along the northern coast of Chile.

### 4.3 Gonadal histological description in females

The TL of the female specimens ranged from 43.0 to 49.4 cm. Based on the gonadal maturity scale proposed by Brown-Peterson *et al*., ^29^ only two of the six maturation stages were observed: inactive and previtellogenic. Among the 224 females captured, only 31 exhibited the previtellogenic stage.

The absence of mature females in this study is consistent with the findings of Barrett, ^16^ who reported that *S. chiliensis* females from the Chilean coast reach sexual maturity at a TL of 51 cm. Similarly, Chirinos de Vildoso ^12^ observed that Peruvian *S. chiliensis* females begin spawning at lengths between 47 and 53 cm. These observations also align with the findings of Goldberg and Mussiett, ^20^ who recorded the smallest spawning female at 48.5 cm TL, captured off the coast of Iquique between November 1981 and December 1982. Estimation of the length at first sexual maturity (L50) is an important parameter in fisheries biology and stock assessment. However, the present dataset primarily contained individuals at early gonadal stages and did not include mature females. Consequently, it was not possible to estimate the size at first maturity for *S. chiliensis*. Future research incorporating a broader size range and all maturity stages would be required to determine this parameter accurately.

An important aspect of this study is the absence of females in advanced maturation stages (full vitellogenesis, hydration, or spawning). This limitation, associated with the sampling period, explains why only two female gonadal stages (inactive and previtellogenic) were described. We recognize that this situation restricts the possibility of establishing a complete histological maturity scale for the species. However, these results should be understood as a preliminary step that provides the first histological characterization of the initial maturation stages in female *S. chiliensis* from northern Chile, laying the groundwork for future studies with broader temporal coverage. The absence of mature females in the sampled population likely reflects the temporal and spatial limitations of the sampling design rather than the absence of reproductive activity in the species.

The temperature is a key factor influencing the ovarian cycle of *S. chiliensis*, which may explain why 47 cm is often reported as the minimum length for sexual maturity. ^47^ In this study, however, the largest previtellogenic female measured 49.4 cm—slightly larger than the smallest spawning female (48.5 cm) reported by Goldberg and Mussiett. ^20^ This discrepancy may be explained by environmental conditions. The fish examined by Goldberg and Mussiett were collected during an “El Niño” event, which caused sea surface temperatures to rise by more than 8°C. This unusual warming likely influenced early gonadal maturation, particularly in northern Chile. ^48^

In contrast, the specimens in this study were collected between December 2013 and June 2014 along the coastal margin of the Chanavayita sector, approximately 60 km south of Iquique in the Tarapacá Region. This period coincided with a strong La Niña event and intense coastal upwelling, both of which contributed to lower-than-average water temperatures. ^49^ The incomplete gonadal development observed in females may indicate temperature-driven segregation—a factor not accounted for in the sampling design of this study.

Although this research did not capture specimens at all stages of ovarian development, thereby preventing the characterization of a complete gonadal maturity scale, it enabled a microscopic description of two key stages from the Brown-Peterson *et al*. ^29^ scale: inactive maturation and previtellogenic maturation. For *S. chiliensis*, Gálvez and Castillo ^3^ adopted the methodology of Kjesbu *et al*. ^25^ to define six distinct stages of female gonadal maturation, integrating both macroscopic and microscopic criteria. Future research could investigate how these stages correspond to the classification proposed by Brown-Peterson *et al*. ^29^

Microscopic examination of inactive female gonads, stained with H&E, revealed an external layer composed of dense CT with uniformly arranged collagen fibers. These fibers extended toward the medullary zone of the gland in thinner bundles, forming lobular structures. This anatomical organization corresponds to the virginal gonadal stage previously described by Gálvez and Castillo ^3^ in females collected from Peruvian waters.

The results demonstrated that the lamellae were arranged in rows, containing numerous immature oocytes and few, if any, PvO. Additionally, empty spaces were observed between the lamellae. Although information on oocyte maturation in *S. chiliensis* remains limited, the findings of this study closely resemble those reported by Brown-Peterson *et al*. ^29^ for *Graus nigra.* Notable similarities include the presence of early perinucleolar stage oocytes (oocyte I), characterized by a centrally located nucleus with multiple NO migrating toward the periphery of the nucleoplasm. Once this migration begins, the nucleus is referred to as the germinal vesicle. ^50– 52^

While no peripheral cytological structural changes were observed, significant alterations occurred in the medullary zone, primarily due to an increase in oocyte size (250–300 μm). The oocytes varied in shape, ranging from round to ovoid, and exhibited centrally positioned ovoid nuclei. The cytoplasm remained homogeneous but displayed reduced basophilia compared to earlier stages. Additionally, cytoplasmic vesicles associated with early oocyte development were observed surrounding the nucleus, consistent with early stages of ovarian development and thereby marking the pre-vitellogenic stage. PvO represent early phases of ovarian development and are commonly present in immature females or during periods of reproductive quiescence. Therefore, their presence should not be interpreted as evidence of active vitellogenesis or imminent spawning activity.

The CT surrounding the gland exhibited an increase in collagenous fibers arranged uniformly—an indicator characteristic of the resting stage (Stage I), as described by Gálvez and Castillo. ^3^ At this stage, oocytes II lacked follicular cells and were instead enclosed by CT, which extended into the medullary zone in thinner bundles, forming lobular structures.

According to Vazzoler, ^53^ the formation and accumulation of yolk is one of the most critical processes in oocyte maturation. However, this process was not observed in the present study.

### 4.4 Gonadal histological description in males

The gonadal stages of *S. chiliensis* males were classified as immature or mature, consistent with the findings of Brown-Peterson *et al*. ^29^ Unlike females, males exhibited asynchronous maturation, meaning they could attain full maturity at any time of the year. This pattern was observed in the present study. Of the 205 male specimens analyzed, 51% were sexually mature, supporting the observations of Barrett ^16^ and Chirinos de Vildoso, ^12^ who identified male maturation as a prerequisite for the spawning period of *S. chiliensis.*

The TL of captured males ranged from 42.6 to 49.0 cm, with both immature and mature individuals represented. Immature males measured between 42.6 and 48.5 cm, while mature males ranged from 45.5 to 49.0 cm. The mature specimens in this study were smaller than those described by Barrett, ^16^ who reported sperm saccules filled with SZ in larger individuals. Gálvez and Castillo ^3^ proposed a five-stage classification system for males, ranging from Stage 0 to Stage IV, based on criteria such as blood supply, sperm fluid flow, CT structure, and germ cell development.

Immature males in the present study corresponded to the Virginal (Stage 0) and Resting (Stage I) categories of Gálvez and Castillo. ^3^ Immature gonads were smaller than mature ones and contained CT extending toward the medullary zone. The cortical and medullary structures of these gonads consisted of continuous cellular cords, within which SZ were absent, including in the SD. Only developing SZ were observed.

Mature specimens corresponded to the Mature (Stage II) and Mature (Stage III) classifications of Gálvez and Castillo. ^3^ These gonads exhibited slight cortical immaturity, with an albuginea composed of CT and collagen fibers. The CT extended into the medullary zone, forming saccules arranged concentrically. The central gonad contained the MSD, composed of smooth muscle interspersed with collagen fibers and lined by a simple flat epithelium. A large quantity of SZ was present within. In each saccule, SG characteristic of spermatogenesis were observed in one half, while the other half contained mature SZ.

No specimens were classified as Expeller (Stage IV), as no empty spaces were observed in the collecting tubes—an indicator of sperm expulsion. The lower SZ count in these specimens may be related to their smaller size (maximum 49.0 cm TL in this study) compared with the 64 cm TL reported by Gálvez and Castillo. ^3^ The terminology and description of spermatogenic development in *S. chiliensis* are consistent with observations in other teleosts. ^54^

Gonadal maturity scales should be integrated with reproductive indices, such as the GSI, fat content, and condition factor. ^25, 26^ Additionally, measurements of oocyte diameter ^55^ and blood hormone levels ^56^ can further refine assessments of reproductive status.

### 4.5 Potential for spawning migrations

Although specific data on the reproductive migratory movements of this species are limited, some historical evidence suggests differentiated spatial patterns during the breeding season. For instance, Serra *et al.* ^17^ and Barrett ^16^ reported that during the breeding months (austral spring–summer), *S. chiliensis* tends to concentrate in certain coastal areas, suggesting movements toward optimal spawning habitats, such as warmer waters or sheltered regions. Similarly, Chirinos de Vildoso ^12^ in Peru documented seasonal changes in the distribution of *S. chiliensis* associated with reproductive activity, indicating shifts toward preferred spawning grounds.

For other species within the genus *Sarda*, such as *Sarda sarda sarda* in the Atlantic, some authors have described a coastal reproductive migration strategy in which adults approach the coast to spawn in warmer, shallower waters. ^57^ These findings support the hypothesis that *S. chiliensis* may exhibit similar behavior, migrating from distant feeding areas to specific spawning locations that are not necessarily accessible from the artisanal fishing zones where our sampling was conducted.

The absence of sexually mature females among the sampled specimens may be attributed, at least in part, to spatial migration to as-yet unidentified spawning areas—a critical aspect for understanding the reproductive dynamics and population structure of this species. ^58^

### 4.6 Development perspective for marine fish aquaculture

The results of this study represent a first step toward the histological characterization of the gonads of *S. chiliensis* in northern Chile. We acknowledge that the descriptive nature of this work and the limited temporal coverage restrict the possibility of directly extrapolating conclusions to aquaculture. Nevertheless, the identification of early gonadal stages under natural conditions provides a fundamental reference for future research aimed at reproductive control in captivity.

To advance in this line, it will be necessary to extend the sampling period to cover at least a full annual cycle, which would allow for a more accurate documentation of the onset of vitellogenesis, spawning events, and post-reproductive regression phases. Likewise, expanding the spatial coverage of sampling across the species’ latitudinal range, from northern Peru to southern Chile, will make it possible to explore regional variations in reproductive dynamics associated with thermal and oceanographic gradients.

Furthermore, the use of quantitative histological parameters—such as seminiferous tubule diameter, germinal epithelium thickness, and cell density—combined with qualitative descriptions, will provide greater precision and reproducibility in the classification of maturity stages, particularly in males. This information will not only contribute to completing the gonadal maturity scale for the species but also generate valuable inputs for fisheries management and conservation, with potential applications in domestication and marine aquaculture programs. Broodstock management should focus on genetic selection and conditioning in controlled environments to synchronize sexual maturation. ^59^

In this context, the results presented here should be understood as an indispensable starting point and not as a conclusive framework for the direct implementation of aquaculture strategies. Their value lies in establishing a reference baseline for future, broader experimental studies that integrate physiological, environmental, and technological aspects required to effectively evaluate the feasibility of controlled reproduction of *S. chiliensis* in culture systems.

Accordingly, any application of these results to aquaculture should be interpreted as a preliminary framework and not as established protocols, which can only be developed through more extensive and experimental research.

### 4.7 Projections for future research

The results of this study represent a first step toward characterizing the gonads of *S. chiliensis* in northern Chile. However, to achieve a more comprehensive understanding of the species’ reproductive cycle, future research with a broader scope is necessary. First, the sampling period should be extended to cover at least one full annual cycle, encompassing different seasons to more accurately document the complete sequence of gonadal stages, including the onset of vitellogenesis, spawning events, and post-reproductive regression stages. ^60^ Second, it is recommended to increase sampling campaigns across the species’ latitudinal range, including northern Peru as well as central and southern Chile. This spatial expansion would allow for the analysis of potential geographic variations in reproductive seasonality related to regional thermal or oceanographic gradients, thereby improving understanding of population connectivity and differentiation. ^61^

Additionally, to achieve a more precise and standardized classification of gonadal maturity stages, particularly in males, quantitative histological parameters—such as the diameter of seminiferous tubules, the thickness of the germinal epithelium, and cell density ^62^—should be employed. When combined with qualitative observations, these parameters would allow for a more reproducible description of spermatogenic development and establish a standard for using appropriate histological characters in commercially important pelagic fish. This approach will contribute not only to completing the scale of gonadal maturity for the species but also to generating valuable information for fishery management and resource conservation, with potential applications in marine aquaculture. The histological observations obtained in this study provide baseline information on the early stages of gonadal development in *S. chiliensis*. Females were predominantly observed in inactive and previtellogenic stages, while males presented immature and mature testes. These findings suggest that the sampled population was primarily composed of individuals in early reproductive phases during the study period, highlighting the importance of extended seasonal sampling to capture the full sequence of gonadal maturation. Despite these limitations, the histological observations obtained in this study provide baseline information on gonadal structure and early developmental stages in *S. chiliensis*. Such descriptive data are important for establishing reference information that can support future reproductive studies, including investigations on spawning dynamics, reproductive seasonality, and stock assessment. Baseline reproductive descriptions are particularly valuable for species in which detailed reproductive histology has not been extensively documented for specific geographic regions.

This study offers a mainly descriptive approach focused on the histological characterization of the early gonadal stages of *S. chiliensis.* We recognize this limitation; however, this information represents a fundamental reference for future research with a broader experimental scope. Accurate identification of the early stages of gonadal maturation is critical to complete the maturity scales of this species, thereby improving fisheries management models, protecting critical reproductive periods, and supporting the development of captive breeding strategies for aquaculture. In this context, the results presented here constitute an indispensable starting point for both the conservation and sustainable exploitation of *S. chiliensis* in the region. Baseline information on gonadal development is essential for fisheries management because it contributes to the identification of reproductive periods and the protection of spawning stocks. Although the present study focuses on early gonadal stages, the results provide valuable reference data for monitoring reproductive conditions in *S. chiliensis*. Such information may support future management strategies, including the identification of potential reproductive periods, the design of seasonal fishing regulations, and the development of stock assessment frameworks for this commercially important species.

## 5. Conclusions

Due to the fact that sampling was limited to the December–June period of a single year, this study should be understood as a preliminary contribution that provides initial histological information on the early gonadal stages of *S. chiliensis* in northern Chile, laying the groundwork for future studies of longer temporal duration and broader spatial scope.

In the absence of mature females, this work should be considered a preliminary approach that describes only the initial stages of gonadal maturation in female *S. chiliensis*, constituting a reference contribution that must be complemented by future research aimed at covering a complete reproductive cycle.

## Ethical considerations

Ethical standards were maintained, as animals were obtained from the city’s fishing terminal shortly after being caught by artisanal fishermen. Legally, *S. chiliensis* is not subject to catch restrictions or protection as an endangered species.

## AI use disclosure

During the preparation of this work the authors used Scopus AI to retrieve scientific literature, and Paperpal and Chat GPT 5.0 in order to correct grammar. After using this tool, the authors reviewed and edited the content as needed and take(s) full responsibility for the content of the publication.

## Data Availability

The data that support the findings of this study (morphometrics, GSI values, maturity stages and Fulton’s condition factor) are openly available from GitHub at: https://github.com/jordan19921992/Raw_data (https://doi.org/XXXXX). ^63^ This dataset was made available under the Creative Commons Attribution 4.0 International (CC BY 4.0) license.
