# Supplementary material for: First Gonadal Stages of Bonito, Sarda chiliensis (Perciformes: Scombridae) on the Pacific Coast of Northern Chile
Source: F1000Res. 2026 Apr 24;14:1346. Originally published 2025 Dec 1. [Version 2] doi: 10.12688/f1000research.173271.2 (PMC13218412; doi:10.12688/f1000research.173271.2)
Supplement: Peer review history

# Reviewer report: Reviewer response for version 2

**DOI:** 10.5256/f1000research.197464.r478859

**Published:** 2026-05-11

**Authors:** Md. Mahiuddin Zahangir

**Affiliations:** 1 Chattogram Veterinary and Animal Sciences University, Chattogram, Bangladesh

The authors have addressed almost all the comments raised by the reviewer. Therefore, I don't have any further comments for this manuscript.

Is the work clearly and accurately presented and does it cite the current literature?

Yes

If applicable, is the statistical analysis and its interpretation appropriate?

Yes

Are all the source data underlying the results available to ensure full reproducibility?

Partly

Is the study design appropriate and is the work technically sound?

Yes

Are the conclusions drawn adequately supported by the results?

Yes

Are sufficient details of methods and analysis provided to allow replication by others?

Yes

Reviewer Expertise:

Reproductive biology, Fish physiology, Climate change and Aquaculture

I confirm that I have read this submission and believe that I have an appropriate level of expertise to confirm that it is of an acceptable scientific standard.

# Reviewer report: Reviewer response for version 2

**DOI:** 10.5256/f1000research.197464.r478858

**Published:** 2026-05-06

**Authors:** Constantinos C Mylonas

**Affiliations:** 1 Hellenic Centre for Marine Research, Heraklion, Greece

As I mentioned in my first report, the study does not achieve the stated objective, which is to obtain reproduction related data important in the proper stock management of this species, such as age at first maturity, reproductive season and description of gametogenesis in both sexes.  Unfortunately, perhaps due to erroneous sampling area and/or sampling time, the authors failed to collect the proper (types) samples, and no reproductively mature females were collected. 

 There is no such thing as “a study of early stages of oogenesis” for fisheries purposes!  The samples collected were simply not in reproductive development.  So, the study does not offer anything in regards to the females.  Preliminary data, should be followed with proper data that will advance our knowledge, not simply fill the scientific literature with data that have no usefulness.  As I said in my first evaluation, the authors should first complete the study and then attempt to publish their results.  The data provided by this manuscript does not advance our understanding of the reproductive biology of the species, nor does it offer any useful information for fisheries management or broodstock conditioning.

Is the work clearly and accurately presented and does it cite the current literature?

No

If applicable, is the statistical analysis and its interpretation appropriate?

No

Are all the source data underlying the results available to ensure full reproducibility?

Yes

Is the study design appropriate and is the work technically sound?

No

Are the conclusions drawn adequately supported by the results?

No

Are sufficient details of methods and analysis provided to allow replication by others?

No

Reviewer Expertise:

Fish reproduction, broodstock management in aquaculture, assisted reproduction technologies

I confirm that I have read this submission and believe that I have an appropriate level of expertise to state that I do not consider it to be of an acceptable scientific standard, for reasons outlined above.

# Reviewer report: Reviewer response for version 1

**DOI:** 10.5256/f1000research.191067.r442254

**Published:** 2026-01-29

**Authors:** Md. Mahiuddin Zahangir

**Affiliations:** 1 Chattogram Veterinary and Animal Sciences University, Chattogram, Bangladesh

This manuscript is about the gonadal stages of Bonito, and the authors have presented data on the condition factor, GSI and gonadal histology from both male and female, which confirms the spawning season. However, information regarding the environmental synchrony with the spawning periodicity, length at first sexual maturity, and the relationship between gastrointestinal indices and the GSI is still missing. The authors are requested to compile the data and propose a detailed overview of the gonadal maturation of the studied species. It is also necessary to describe how the current data can be used to undertake policy management for this species.

Is the work clearly and accurately presented and does it cite the current literature?

Yes

If applicable, is the statistical analysis and its interpretation appropriate?

Yes

Are all the source data underlying the results available to ensure full reproducibility?

Partly

Is the study design appropriate and is the work technically sound?

Yes

Are the conclusions drawn adequately supported by the results?

Yes

Are sufficient details of methods and analysis provided to allow replication by others?

Yes

Reviewer Expertise:

Reproductive biology, Fish physiology, Climate change and Aquaculture

I confirm that I have read this submission and believe that I have an appropriate level of expertise to confirm that it is of an acceptable scientific standard, however I have significant reservations, as outlined above.

# Response

**Published:** 2026-03-11

**Authors:** Jordan Ismael Huanacuni Pilco

**Affiliations:** Tacna, Universidad Tecnológica del Perú, Tacna, Tacna, Peru

**Response to Reviewer 3**

  Approved With Reservations

 This manuscript is about the gonadal stages of Bonito, and the authors have presented data on the condition factor, GSI and gonadal histology from both male and female, which confirms the spawning season.

 Dear Reviewer,

 We sincerely thank you for your constructive comments and for recognizing the contribution of our study to the understanding of gonadal development in *Sarda chiliensis*. We appreciate the suggestions aimed at strengthening the ecological and management context of the manuscript. In response to your comments, we have expanded the Discussion section to clarify the limitations of the current dataset and to provide a broader overview of gonadal maturation patterns, environmental influences, and potential implications for fisheries management. Our detailed responses are provided below.

**Comment 1**

*Information regarding environmental synchrony with spawning periodicity is missing.*

**Response:**

 We agree that environmental variables may play an important role in regulating reproductive cycles in pelagic fishes. However, the temporal scope of our sampling did not allow us to fully evaluate environmental synchrony with spawning periodicity. To address this point, we have expanded the Discussion to acknowledge this limitation and to highlight the importance of future studies integrating environmental monitoring and reproductive indicators.

 We have added the following to the end of the first paragraph of subsection 4.1. Gonadosomatic Index (GSI)

*Reproductive cycles in pelagic fishes are often influenced by environmental variability, including temperature fluctuations, food availability, and oceanographic processes. Although the present study recorded environmental parameters during the sampling period, the temporal coverage of the dataset was insufficient to evaluate potential synchrony between environmental variability and spawning periodicity in* *Sarda chiliensis* *. Future studies incorporating multi-year sampling and continuous environmental monitoring would provide a more comprehensive understanding of the environmental drivers of reproduction in this species.*

**Comment 2**

*Information on length at first sexual maturity is missing.*

**Response:**

 We appreciate this suggestion. Estimating the length at first sexual maturity requires the presence of fully mature individuals across multiple maturity stages. Because our dataset included primarily early gonadal stages and did not contain mature females, it was not possible to estimate this parameter reliably. We have clarified this limitation in the Discussion.

 We have added the following information at the end of the second paragraph of subsection 4.3 Gonadal histological description in females

*Estimation of the length at first sexual maturity (L50) is an important parameter in fisheries biology and stock assessment. However, the present dataset primarily contained individuals at early gonadal stages and did not include mature females. Consequently, it was not possible to estimate the size at first maturity for* *S. chiliensis* *. Future research incorporating a broader size range and all maturity stages would be required to determine this parameter accurately.*

**Comment 3**

*Relationship between gastrointestinal indices and GSI.*

**Response:**

 We thank the reviewer for this suggestion. Unfortunately, gastrointestinal indices were not systematically recorded during the sampling process. Therefore, such analyses were beyond the scope of the present study. This limitation is now acknowledged in the Discussion.

 We have added the following information at the end of the second paragraph of subsection 4.1 Gonadosomatic Index (GSI)

*The relationship between feeding activity and reproductive development can provide insights into the energetic allocation strategies of fish species. In the present study, gastrointestinal indices were not systematically recorded during sampling, which prevented evaluation of potential relationships between feeding intensity and gonadal development. Incorporating trophic indicators in future studies would allow further exploration of the interaction between feeding dynamics and reproduction.*

**Comment 4**

*The authors are requested to compile the data and propose a detailed overview of the gonadal maturation of the studied species.*

**Response:**

 We appreciate this recommendation. The Discussion section has been expanded to summarize the main features of gonadal development observed in the analyzed specimens and to contextualize them with previous reproductive studies of pelagic fishes.

 We have added the following information at the end of the second paragraph of subsection 4.7 Projections for future research

 The histological observations obtained in this study provide baseline information on the early stages of gonadal development in *S. chiliensis*. Females were predominantly observed in inactive and previtellogenic stages, while males presented immature and mature testes. These findings suggest that the sampled population was primarily composed of individuals in early reproductive phases during the study period, highlighting the importance of extended seasonal sampling to capture the full sequence of gonadal maturation.

**Comment 5**

*It is also necessary to describe how the current data can be used to undertake policy management for this species*****

**Response:**

 We agree that reproductive information is valuable for fisheries management. We have therefore incorporated a paragraph discussing how baseline information on gonadal development can support fisheries monitoring, reproductive protection measures, and future stock assessment studies.

 We have added the following information at the end of the of subsection 4.7 Projections for future research

 Baseline information on gonadal development is essential for fisheries management because it contributes to the identification of reproductive periods and the protection of spawning stocks. Although the present study focuses on early gonadal stages, the results provide valuable reference data for monitoring reproductive conditions in *S. chiliensis*. Such information may support future management strategies, including the identification of potential reproductive periods, the design of seasonal fishing regulations, and the development of stock assessment frameworks for this commercially important species.

# Reviewer report: Reviewer response for version 1

**DOI:** 10.5256/f1000research.191067.r444583

**Published:** 2026-01-13

**Authors:** Constantinos C Mylonas

**Affiliations:** 1 Hellenic Centre for Marine Research, Heraklion, Greece

The manuscript presents a study of the gametogenesis of an important fishery species for South America, the bonito ( *Sarda chiliensis*).  Members of this genus are very important pelagic fish, and they are highly sought by the world fishing fleet.  The stated objective of the authors, is to obtain reproduction related data, that is important in the proper stock management of this species, such as age at first maturity, reproductive season and description of the proves of gametogenesis in both sexes.  Unfortunately, the authors failed to collect the proper (types) samples, they did not make the appropriate statistical analyses (size, GSI, maturity) and focused almost exclusively on presenting histological methods.  The authors claim that their study should “not be interpreted as confirmatory evidence, but rather as a first step to guide more comprehensive analyses in future research”.  Data obtained from Incomplete or inappropriate experimental designs should be indexed as “preliminary”, but the authors should first complete the study and then attempt to index their results.  The data provided by this manuscript does not advance our understanding of the reproductive biology of the species, nor does it offer any useful information for fisheries management or broodstock conditioning.  The stages of gametogenesis in fish are well know for decades now, and there is no reason to expect that this species is any different from other finfish of the same order or family.  Specific criticisms follow.

 The authors collected samples from the fish market during the period between December 2013 and June 2014.  As such, they inherently **did not include the annual cycle of the fish**.  This is perhaps one (the main?) reason why they did not find any reproductively active or mature females.  Then, the samples were presumably from the area of Chanavita, but this is probably not quite certain, as I assume they relied on the claims of the fishermen.  Collecting fish from a single area may mean that this is not a reproductive ground for the fish, and then this may be another reason why they did not find any mature females.  Another, perhaps less important aspect that makes this study less significant, is that the samples were taken by the authors and processed presumably 7 hrs after capture by the fishermen, something that again may not be certain.  And no information on how the fish were stored during these 7 hrs, prior to processing, which may result in artifacts in the histological sections that are not related to the proper functioning of gametogenesis. 

 No statistical analysis seems to have been made (at least there is no report of the type of analysis that was done) of the total length or body weight of males vs females, or of mature vs immature (males), or Previtellogenic vs Immature (females).  Such analysis will give some information on whether size made a difference in the reproductive stage of the fish.  This is important information in all fish species.

 Regarding the absence of mature fish, the author seems to claim that because there was no breeding behavior, this resulted in the absence of mature females.  This interpretation is incorrect and a bit flippant, since breeding behaviors follow reproductive maturation and not the other way around.

 Regarding the claimed “…monthly variation in GSI..” (Section 4.1), there is no evidence in the manuscript (Fig. 2) of such a variation, and as I pointed earlier, no such statistical analysis is shown.

 Finally, the oocyte stage that the authors claim as “pre vitellogenesis”, is well known to be a stage that is not-sensitive to the gonadotropin hormones, and it does not indicate that the fish have entered the process of gametogenesis (as the term pre-, correctly suggests).  As a result, when we follow the gametogenic cycle, we do not use the presence of a very limited number of oocytes in the Cortical Alveoli or Lipid vesicle stage, as an indication of the initiation of vitellogenesis and the development towards maturity.  Immature females often have some of these oocytes throughout the period of quiescence (pre-vitellogenesis), and it cannot usually help us in defining the period of gametogenesis.

 Figure 2.  What is a “dormant desired” stage?  I assume it is referring to a female?  Why isn’t there a statistical analysis of the GSI data?  As the authors mentioned, this parameter is the main one used by older studies and by fisheries scientists to describe the reproductive status of fish, and it is necessary to document if (a) there are differences between males and females, (b) immature and mature individuals, and (c) during the annual reproductive season.  This information is missing for the manuscript.

 Table 3. The authors report “nucleoli at the periphery” of PvO oocytes, and refer to Fig 6a and b.  Firstly, nucleoli are in the periphery of the nuclei, not of the oocytes (!) and secondly, I do not see any “vesicles” in the cytoplasm of Figure 6a and b.

 Figure 6.   In photo “f”, I do not agree that what is shown is a “nucleolus”, which is a sub-cellular structure within the nucleus of an oocyte!  In fact, right next to this structure, one can see a few primary oocytes (po) correctly stained dark and uniformly purple, and these are manifold smaller than this supposed “nucleolus”.  Also, within some of these primary oocytes, one can see their nuclei, which stain a bit lighter.

 Figure 8.  The histological photos are of poor quality, and one cannot really see the details of the section.  Higher magnification should be used, so than the difference between immature and mature, spermatozoa containing testes can be clearly distinguished.

 Figure 9.  What is a “spermatogenic saccule” (perhaps spermatocysts?).  I have never seen this term before, and the authors should examine more recent articles that use the correct terminology, as I indicated earlier. Also, in Fig 9a, there is a black arrow that is not cited in the legend.  To what is it pointing”. Conversely, there is a mention of a white arrow, which is not visible.

 The authors should read and use the terminology presented more recently (Brown-Peterson et al., 2011), as the one used (Oliva J, 1968) is too old, not in English and, therefore, not widely available, accessible and readable to contemporary researchers.  We should all strive to use conventional terminologies, so that our work can be properly evaluated and used by our peers.

 Bibliography:

 Reference 1

 Reference 2

Is the work clearly and accurately presented and does it cite the current literature?

No

If applicable, is the statistical analysis and its interpretation appropriate?

No

Are all the source data underlying the results available to ensure full reproducibility?

Yes

Is the study design appropriate and is the work technically sound?

No

Are the conclusions drawn adequately supported by the results?

No

Are sufficient details of methods and analysis provided to allow replication by others?

No

Reviewer Expertise:

Fish reproduction, broodstock management in aquaculture, assisted reproduction technologies

I confirm that I have read this submission and believe that I have an appropriate level of expertise to state that I do not consider it to be of an acceptable scientific standard, for reasons outlined above.

## References

[1] : A Standardized Terminology for Describing Reproductive Development in Fishes. *Marine and Coastal Fisheries* .2011;3(1) : 10.1080/19425120.2011.555724 52-70 10.1080/19425120.2011.555724

[2] : Prosodic Biología reproductiva e histología de gónadas en peces. *https://www.researchgate.net/publication/228809041_Prosodic_parameters_for_the_detection_of_regiona…* .

# Response

**Published:** 2026-03-13

**Authors:** Jordan Ismael Huanacuni Pilco

**Affiliations:** Tacna, Universidad Tecnológica del Perú, Tacna, Tacna, Peru

**REVIEWER 2**

**Comment**

 The manuscript presents a study of the gametogenesis of an important fishery species for South America, the bonito ( *Sarda chiliensis*).  Members of this genus are very important pelagic fish, and they are highly sought by the world fishing fleet.  The stated objective of the authors, is to obtain reproduction related data, that is important in the proper stock management of this species, such as age at first maturity, reproductive season and description of the proves of gametogenesis in both sexes.  Unfortunately, the authors failed to collect the proper (types) samples, they did not make the appropriate statistical analyses (size, GSI, maturity) and focused almost exclusively on presenting histological methods.  The authors claim that their study should “not be interpreted as confirmatory evidence, but rather as a first step to guide more comprehensive analyses in future research”.  Data obtained from Incomplete or inappropriate experimental designs should be indexed as “preliminary”, but the authors should first complete the study and then attempt to index their results.  The data provided by this manuscript does not advance our understanding of the reproductive biology of the species, nor does it offer any useful information for fisheries management or broodstock conditioning.  The stages of gametogenesis in fish are well know for decades now, and there is no reason to expect that this species is any different from other finfish of the same order or family.  Specific criticisms follow.

**Response:**

 Dear Reviewer, we sincerely thank you for the time and expertise dedicated to evaluating our manuscript. We appreciate your detailed and critical comments, which helped us clarify the scope of the study and improve the presentation of our results.

 We would like to emphasize that the primary objective of this work was to provide a histological description of the early stages of gonadal development in *Sarda chiliensis* based on the available samples. We recognize the limitations of the sampling design and have revised the manuscript to clearly state that the results should be interpreted as baseline descriptive information rather than a complete characterization of the reproductive cycle.

 Below we address each of your comments individually and describe the modifications incorporated into the revised manuscript.

**Q1. **The authors collected samples from the fish market during the period between December 2013 and June 2014.  As such, they inherently did not include the annual cycle of the fish.  This is perhaps one (the main?) reason why they did not find any reproductively active or mature females.  Then, the samples were presumably from the area of Chanavita, but this is probably not quite certain, as I assume they relied on the claims of the fishermen.  Collecting fish from a single area may mean that this is not a reproductive ground for the fish, and then this may be another reason why they did not find any mature females.  Another, perhaps less important aspect that makes this study less significant, is that the samples were taken by the authors and processed presumably 7 hrs after capture by the fishermen, something that again may not be certain.  And no information on how the fish were stored during these 7 hrs, prior to processing, which may result in artifacts in the histological sections that are not related to the proper functioning of gametogenesis. 

**Response**

 We thank for this important comment. We agree that the sampling period does not represent the complete annual reproductive cycle of *Sarda chiliensis*. The objective of the present study was not to characterize the full reproductive cycle but rather to document the gonadal stages observed in the available specimens during the sampling period.

 The manuscript has been revised to clarify this scope and explicitly acknowledge the temporal limitations of the dataset.

 Regarding the sampling procedure, the specimens were obtained from artisanal fishery landings and processed shortly after arrival at the fish market. Although this approach does not allow independent verification of the exact capture location, market-based sampling is commonly used in fisheries biology when onboard sampling is not feasible, particularly for commercially exploited pelagic species.

 To minimize potential histological artifacts, the fish were transported on ice and processed within a few hours after landing prior to fixation.

**Q2. **No statistical analysis seems to have been made (at least there is no report of the type of analysis that was done) of the total length or body weight of males vs females, or of mature vs immature (males), or Previtellogenic vs Immature (females).  Such analysis will give some information on whether size made a difference in the reproductive stage of the fish.  This is important information in all fish species.

**Response**

 We appreciate the suggestion regarding morphometric comparisons among sexes and maturity stages. Additional descriptive analyses were incorporated to summarize total length, body weight, gonadosomatic index (GSI), and Fulton’s condition factor for males and females.

 Because the dataset included a limited number of maturity stages, particularly in females, these analyses were performed as descriptive summaries rather than formal statistical comparisons. The purpose of including these analyses is to provide an overview of the biological characteristics of the sampled individuals and to facilitate future comparative studies.

**Q3. **Regarding the absence of mature fish, the author seems to claim that because there was no breeding behavior, this resulted in the absence of mature females.  This interpretation is incorrect and a bit flippant, since breeding behaviors follow reproductive maturation and not the other way around.

**Response**

 We thank for this important clarification. We agree that reproductive maturation precedes spawning behavior and that the absence of observed breeding behavior cannot explain the lack of mature females in the sampled individuals. The sentence suggesting this interpretation has been removed from the manuscript and replaced with a more appropriate explanation emphasizing the temporal and spatial limitations of the sampling design.

 We have added the following text at the end of the third paragraph of section 4.3:

*The absence of mature females in the sampled population likely reflects the temporal and spatial limitations of the sampling design rather than the absence of reproductive activity in the species.*

**Q4. **Regarding the claimed “…monthly variation in GSI..” (Section 4.1), there is no evidence in the manuscript (Fig. 2) of such a variation, and as I pointed earlier, no such statistical analysis is shown.

**Response**

 We appreciate the  observation. We agree that the available data do not support a statistical evaluation of monthly variation in GSI. The wording in the manuscript has therefore been revised to avoid implying statistically supported temporal variation and to clarify that the observed differences represent descriptive fluctuations within the sampling period.

 We have included the following sentence in the first paragraph of section 4.1.

*The* *GSI values showed fluctuations during the sampling months; however, given the limited temporal coverage and sample distribution, these observations should be interpreted cautiously.*

**Q5.** Finally, the oocyte stage that the authors claim as “pre vitellogenesis”, is well known to be a stage that is not-sensitive to the gonadotropin hormones, and it does not indicate that the fish have entered the process of gametogenesis (as the term pre-, correctly suggests).  As a result, when we follow the gametogenic cycle, we do not use the presence of a very limited number of oocytes in the Cortical Alveoli or Lipid vesicle stage, as an indication of the initiation of vitellogenesis and the development towards maturity.  Immature females often have some of these oocytes throughout the period of quiescence (pre-vitellogenesis), and it cannot usually help us in defining the period of gametogenesis.

**Response**

 We appreciate the reviewer’s clarification regarding the interpretation of previtellogenic oocytes. We agree that these oocytes correspond to early phases of ovarian development and that their presence alone does not necessarily indicate the onset of active vitellogenesis or progression toward reproductive maturation. The manuscript has been revised to clarify this point and to avoid implying that the presence of previtellogenic oocytes reflects the initiation of active gametogenesis.

 We have added the following information at the end of the penultimate paragraph

*PvO* *represent early phases of ovarian development and are commonly present in immature females during periods of reproductive quiescence. Therefore, their presence should not be interpreted as evidence of active vitellogenesis or imminent spawning activity.*

**Q6. **Figure 2.  What is a “dormant desired” stage?  I assume it is referring to a female?  Why isn’t there a statistical analysis of the GSI data?  As the authors mentioned, this parameter is the main one used by older studies and by fisheries scientists to describe the reproductive status of fish, and it is necessary to document if (a) there are differences between males and females, (b) immature and mature individuals, and (c) during the annual reproductive season.  This information is missing for the manuscript.

**Response**

 We thank for identifying this issue. The term “dormant desired” resulted from a typographical error and has been corrected. Additionally, figure labels have been revised for clarity.

 We have amended Figure 2. The term ‘dormant desired’ has been replaced with ‘immature female’.

**Q7. **Table 3. The authors report “nucleoli at the periphery” of PvO oocytes, and refer to Fig 6a and b.  Firstly, nucleoli are in the periphery of the nuclei, not of the oocytes (!) and secondly, I do not see any “vesicles” in the cytoplasm of Figure 6a and b.

**Response**

 We thank for pointing out this imprecision. The text has been corrected to specify that nucleoli are located at the periphery of the nucleus.

 We have corrected the description of the morphology in Table 3:

**Q8.** Figure 6.   In photo “f”, I do not agree that what is shown is a “nucleolus”, which is a sub-cellular structure within the nucleus of an oocyte!  In fact, right next to this structure, one can see a few primary oocytes (po) correctly stained dark and uniformly purple, and these are manifold smaller than this supposed “nucleolus”.  Also, within some of these primary oocytes, one can see their nuclei, which stain a bit lighter.

**Response**

 We appreciate the observation and have revised the labeling of the structure in Figure 6 to avoid misinterpretation. The annotation has been corrected to identify the structure more accurately.

 We have changed to:

*(f ) Positive PAS reaction in early primary oocyte (PO) adjacent to the PvO, indicating the presence of glycoproteins (black arrow).*

**Q9.** Figure 8.  The histological photos are of poor quality, and one cannot really see the details of the section.  Higher magnification should be used, so than the difference between immature and mature, spermatozoa containing testes can be clearly distinguished.

**Response**

 We thank for this valuable observation and for the suggestion to include higher-magnification histological images to better illustrate the cellular structures. We agree that higher magnification can improve the visualization of the differences between immature and mature testes. However, the evaluation of this dataset has already been completed and the original histological image archive does not include additional photographs at higher magnification. Therefore, it was not possible to replace the images with higher-magnification micrographs in the revised manuscript.

**Q10.** Figure 9.  What is a “spermatogenic saccule” (perhaps spermatocysts?).  I have never seen this term before, and the authors should examine more recent articles that use the correct terminology, as I indicated earlier. Also, in Fig 9a, there is a black arrow that is not cited in the legend.  To what is it pointing”. Conversely, there is a mention of a white arrow, which is not visible.

**Response**

 We appreciate this observation. The terminology has been revised to align with current terminology used in reproductive biology of fishes. We deplaced: “spermatogenic saccule” with: “spermatocyst”. We apologise for the error in the caption. It has been corrected to 'black arrowhead' instead of 'white arrowhead'.

**Q11.** The authors should read and use the terminology presented more recently (Brown-Peterson et al., 2011), as the one used (Oliva J, 1968) is too old, not in English and, therefore, not widely available, accessible and readable to contemporary researchers.  We should all strive to use conventional terminologies, so that our work can be properly evaluated and used by our peers.

**Response**

 We agree with that standardized terminology is important for comparability across studies. The manuscript has been revised to harmonize the terminology used for reproductive stages with the standardized terminology proposed by Brown-Peterson et al. (2011).

 We have included at the end of 2.3. Gonadal processing:

*Gonadal development stages were described following the standardized terminology proposed by Brown-Peterson et al. (2011), which is widely used in modern studies of fish reproductive biology.*

# Reviewer report: Reviewer response for version 1

**DOI:** 10.5256/f1000research.191067.r440594

**Published:** 2025-12-30

**Authors:** Darmawan Setia Budi

**Affiliations:** 1 Universitas Airlangga, East Java, Indonesia

**General assessment**

 This manuscript presents a detailed histological description of the early gonadal development of *Sarda chiliensis* collected from the northern coast of Chile. The study provides valuable baseline information, particularly because histological descriptions of early gonadal stages for this species in this region are currently scarce. The work is generally well written, methodologically sound, and clearly structured, and it aligns well with the descriptive and data-driven scope of F1000Research.

 The authors appropriately acknowledge the main limitations of the study, particularly the restricted temporal coverage and the absence of mature females. Overall, the data appear reliable, and the conclusions are supported by the results presented. With minor clarifications and some improvements in presentation and contextualization, this article would represent a useful contribution to fisheries biology and reproductive studies of pelagic fishes.

**Major comments**

1. **Scope and interpretation of results**
  The study focuses exclusively on early gonadal stages (inactive and previtellogenic in females; immature and mature in males). While this is clearly stated, some sections of the Discussion occasionally imply broader conclusions regarding the reproductive cycle of *S. chiliensis*. I recommend further emphasizing that the findings are limited to early stages and should not be extrapolated to define the full reproductive cycle or spawning dynamics.
2. **Sampling design and representativeness**
  Sampling was conducted over a single period (December–June of one year) and from one main fishing area. Although this limitation is acknowledged, it would be useful to explicitly discuss how spatial segregation or seasonal migration of mature females could bias the observed sex and maturity composition, particularly in the Results or Discussion.
3. **Statistical analyses**
  The exploratory statistical analyses (e.g. GSI vs. temperature, condition factor relationships) are appropriately described as non-confirmatory. However, the manuscript would benefit from a clearer justification of why these analyses were included and how readers should interpret their biological relevance given the limited temporal resolution.

**Minor comments**

1. **Terminology consistency**
  Please ensure consistent use of maturity stage terminology throughout the manuscript (e.g. “inactive” vs. “dormant” females) to avoid confusion.
2. **Figures and tables**
  - There is a typographical error in **Figure 2**, where *“madurity”* should be corrected to **“maturity.”**
  - Please standardize the formatting of scale bars in all histological images by adding a space between the numerical value and the unit (e.g. **200 μm** instead of 200μm), and ensure consistency between the scale bars shown in the figures and the corresponding descriptions in the figure captions.
  - The histological figures are generally informative; however, consistent labeling (using uniform scale bars) and formatting across figures would further improve clarity. 
3. **Language and style**
  The manuscript is generally clear and readable. Minor grammatical and stylistic edits would further improve flow, particularly in longer Discussion paragraphs. These issues do not affect the scientific content.
4. **Data availability statement**
  The data availability section is clear. It may be helpful to briefly indicate which variables are included in the shared dataset (e.g. morphometrics, GSI values, maturity stages).

**Recommendations**

 This manuscript provides a solid and carefully documented histological baseline for early gonadal development in *Sarda chiliensis* from northern Chile. The study is methodologically sound, transparently reported, and appropriate for publication in F1000Research. I recommend **indexing after minor revisions**, primarily focused on clarifying the scope of inference, improving consistency, and refining figure presentation.

Is the work clearly and accurately presented and does it cite the current literature?

Yes

If applicable, is the statistical analysis and its interpretation appropriate?

Yes

Are all the source data underlying the results available to ensure full reproducibility?

Yes

Is the study design appropriate and is the work technically sound?

Yes

Are the conclusions drawn adequately supported by the results?

Yes

Are sufficient details of methods and analysis provided to allow replication by others?

Yes

Reviewer Expertise:

Fish reproduction, breeding, and domestication

I confirm that I have read this submission and believe that I have an appropriate level of expertise to confirm that it is of an acceptable scientific standard.

## References

[1] : Maturity determination and its potential application for fish captive breeding. *Animal Reproduction Science* .2026;285: 10.1016/j.anireprosci.2025.108074 10.1016/j.anireprosci.2025.108074 41386067

[2] : Sexual Characteristics of Nemipterus peronii (Valenciennes, 1830) in the Bali Strait. *Egyptian Journal of Aquatic Biology and Fisheries* .2025;29(5) : 10.21608/ejabf.2025.412904.6390 2327-2342 10.21608/ejabf.2025.412904.6390

# Response

**Published:** 2026-03-08

**Authors:** Jordan Ismael Huanacuni Pilco

**Affiliations:** Tacna, Universidad Tecnológica del Perú, Tacna, Tacna, Peru

**Reviewer Report – REVIEWER 1**

**General assessment**

 This manuscript presents a detailed histological description of the early gonadal development of *Sarda chiliensis* collected from the northern coast of Chile. The study provides valuable baseline information, particularly because histological descriptions of early gonadal stages for this species in this region are currently scarce. The work is generally well written, methodologically sound, and clearly structured, and it aligns well with the descriptive and data-driven scope of F1000Research.

 The authors appropriately acknowledge the main limitations of the study, particularly the restricted temporal coverage and the absence of mature females. Overall, the data appear reliable, and the conclusions are supported by the results presented. With minor clarifications and some improvements in presentation and contextualization, this article would represent a useful contribution to fisheries biology and reproductive studies of pelagic fishes.

 Response:

 Dear Reviewer,

 We sincerely thank you for the careful evaluation of our manuscript and for your constructive comments. We appreciate your positive assessment of the methodological rigor and clarity of the study. Your suggestions have helped us improve the manuscript, particularly in clarifying the scope of the results, strengthening the contextual discussion, and improving presentation details. Below we address each comment individually.

**Major comments**

**1. Scope and interpretation of results**

 The study focuses exclusively on early gonadal stages (inactive and previtellogenic in females; immature and mature in males). While this is clearly stated, some sections of the Discussion occasionally imply broader conclusions regarding the reproductive cycle of *S. chiliensis*. I recommend further emphasizing that the findings are limited to early stages and should not be extrapolated to define the full reproductive cycle or spawning dynamics.

**Response:**

 We appreciate this important observation. The intention of this study was strictly to document and describe the early gonadal stages observed in *Sarda chiliensis*. Following the reviewer’s suggestion, we revised several sections of the Discussion to explicitly emphasize that our findings are limited to the early stages of gonadal development and should not be interpreted as describing the full reproductive cycle or spawning dynamics of the species.

 We have added the following to the end of the fifth paragraph of the discussion:

*It is important to note that the present study focused exclusively on the early gonadal stages observed in* *Sarda chiliensis* *. Consequently, the findings should be interpreted as a descriptive characterization of initial gonadal development rather than as a complete representation of the species' reproductive cycle. Additional studies covering a broader temporal range and including mature females would be necessary to fully describe the spawning dynamics and reproductive cycle of this species in the study region.*

**2. Sampling design and representativeness**

 Sampling was conducted over a single period (December–June of one year) and from one main fishing area. Although this limitation is acknowledged, it would be useful to explicitly discuss how spatial segregation or seasonal migration of mature females could bias the observed sex and maturity composition, particularly in the Results or Discussion.

**Response:**

 We thank the reviewer for highlighting this point. We agree that spatial segregation or seasonal movements of mature females may influence the observed maturity composition. To address this, we have expanded the Discussion section to acknowledge that mature females may occur in other areas or seasons not covered by our sampling period, which could explain their absence in the collected specimens.

 We have added the following to the end of the fifth paragraph of the discussion:

*The absence of mature females in the analyzed samples may be related to spatial or seasonal segregation patterns. In many pelagic fish species, reproductive individuals may migrate to specific spawning areas or occupy habitats different from those exploited by local fisheries. Therefore, it is possible that mature females of S. chiliensis occur in other areas or during periods not covered by the present sampling. Future studies incorporating wider spatial and temporal sampling would help clarify the reproductive distribution patterns of this species.*

**3. Statistical analyses**

 The exploratory statistical analyses (e.g. GSI vs. temperature, condition factor relationships) are appropriately described as non-confirmatory. However, the manuscript would benefit from a clearer justification of why these analyses were included and how readers should interpret their biological relevance given the limited temporal resolution.

**Response:**

 We appreciate this suggestion. The exploratory analyses were included to provide preliminary insights into possible relationships between environmental conditions and biological indices. However, we agree that these results should be interpreted cautiously due to the limited temporal coverage. We have clarified this purpose in the Methods and Discussion sections.

 We have added to the end of subsection 2.5 Statistical analyses

*Given the limited temporal coverage of the dataset, these analyses should be interpreted cautiously and are intended primarily to identify potential patterns that may guide future research rather than to establish definitive biological relationships.*

**Minor comments**

**1. Terminology consistency**

 Please ensure consistent use of maturity stage terminology throughout the manuscript (e.g. “inactive” vs. “dormant” females) to avoid confusion.

**Response:**

 Thank you for pointing this out. We carefully reviewed the manuscript and standardized the terminology used to describe gonadal stages throughout the text to ensure consistency. Females were classified as inactive or previtellogenic according to histological characteristics.

**2. Figures and tables**

1.
  - There is a typographical error in **Figure 2**, where *“madurity”* should be corrected to **“maturity.”**
  - Please standardize the formatting of scale bars in all histological images by adding a space between the numerical value and the unit (e.g. **200** **μ** **m** instead of 200μm), and ensure consistency between the scale bars shown in the figures and the corresponding descriptions in the figure captions.
  - The histological figures are generally informative; however, consistent labeling (using uniform scale bars) and formatting across figures would further improve clarity. 

**Response:**

 We thank the reviewer for noticing this error. The typographical error in Figure 2 has been corrected from “madurity” to “maturity.”

 All scale bars now include a space between the numerical value and the unit (e.g., 200 μm), and the formatting has been made consistent across figures and captions

 Histological figures were revised to ensure consistent labeling and formatting throughout the manuscript.

**3. Language and style**

 The manuscript is generally clear and readable. Minor grammatical and stylistic edits would further improve flow, particularly in longer Discussion paragraphs. These issues do not affect the scientific content.

**Response:**

 We appreciate this suggestion and performed an additional revision of the manuscript to improve clarity and readability. Minor grammatical and stylistic edits were implemented throughout the manuscript.

**4. Data availability statement**

 The data availability section is clear. It may be helpful to briefly indicate which variables are included in the shared dataset (e.g. morphometrics, GSI values, maturity stages).

**Response:**

 Thank you for this suggestion. The Data Availability section has been expanded to briefly indicate that the dataset includes morphometrics, GSI values, maturity stages and Fulton’s condition factor.
